# Chain‐Length Regulation by WzzE Is Necessary for, but Genetically Separable From, Cyclic Enterobacterial Common Antigen Synthesis

**DOI:** 10.1111/mmi.70088

**Published:** 2026-06-19

**Authors:** Joseph F. Carr, Yohannes H. Rezenom, Jennifer S. Rudolf, Daniel J. Warzecha, Angela M. Mitchell

**Affiliations:** ^1^ Department of Biology Texas A&M University College Station Texas USA; ^2^ Department of Chemistry Texas A&M University College Station Texas USA

**Keywords:** chain length regulation, enterobacterial common antigen, glycan biosynthesis, gram negative envelope, O antigen, Wzz

## Abstract

Enterobacterial common antigen (ECA) is a conserved glycan that supports intrinsic antibiotic resistance in Enterobacterales. ECA exists in outer membrane diacylglycerol‐phosphate‐ and lipopolysaccharide‐linked forms, and a cyclic periplasmic form (ECA_CYC_). Intriguingly, ECA_CYC_ both affects the outer membrane permeability barrier and functions in regulation of diacylglycerol‐phosphate‐linked ECA. While the length of linear ECA polymers generated by WzyE is regulated by the co‐polymerase WzzE, WzzE is also required for ECA_CYC_ biogenesis and no ECA_CYC_ is synthesized in its absence. To define WzzE functions necessary for ECA_CYC_ biosynthesis, we generated plasmid‐borne *wzzE* mutants in *Escherichia coli* K‐12 and quantified their effects on linear ECA regulation and ECA_CYC_ synthesis, determining that mutations disrupting linear ECA regulation in either transmembrane helix 2 or the periplasmic domain abolished ECA_CYC_ synthesis. Moreover, we identified two mutations residue F104 that differed in ECA_CYC_ abundance despite indistinguishable linear ECA regulation: *wzzE*_F104H_ caused an approximately 2‐fold decrease in ECA_CYC_ abundance, whereas *wzzE*_F104Y_ retained wild‐type abundance. Chromosomal *wzzE* mutants recapitulated this phenotype, demonstrating that these substitutions genetically uncouple levels of ECA_CYC_ synthesis from linear ECA regulation. Thus, although WzzE‐mediated chain‐length regulation is necessary for ECA_CYC_ biogenesis, it is not sufficient.

## Graphical abstract

Here, we demonstrate that polysaccharide chain length regulation plays a distinct role in enabling formation of a cyclic molecule important for the bacterial cell surface. However, this function is necessary, but not sufficient for the cyclic molecule's biosynthesis. By uncovering evidence genetically separating cyclic polysaccharide biosynthesis from chain length regulation, our work uncovers how bacteria fine‐tune surface structures, offering new insight into cell envelope organization and potential vulnerabilities that could be targeted in antimicrobial strategies.

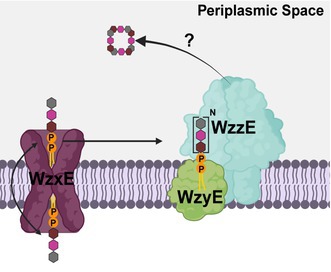

## 1 Introduction

*Enterobacterales* is an order of gram‐negative bacteria that includes numerous genera with human pathogens (e.g., *Escherichia, Salmonella, Klebsiella, Yersinia*) (Doern 2024; Adeolu et al. 2016). These organisms cause a variety of diseases including respiratory, soft tissue, and urinary tract infections, as well as endocarditis (Ramirez and Giron 2024). To survive in their enteric environment, members of this family take advantage of their outer membrane (OM) which acts as a robust permeability barrier composed of an asymmetric bilayer with phospholipids in the inner leaflet and predominantly lipopolysaccharides (LPS) in the outer leaflet (Maher and Hassan 2023; Silhavy et al. 2010). LPS molecules form an extensive hydrophilic network of lateral interactions that excludes many large and hydrophobic compounds—such as many antibiotics, bile salts, and detergents—from entering the cell (Farfour et al. 2022; Aminov 2010).

Beyond LPS, the outer membrane is decorated with other components critical for fitness, including enterobacterial common antigen (ECA) (Hill and Mitchell 2025; Rai and Mitchell 2020). Identified in 1962 by Calvin M. Kunin et al. as a “common antigen” associated with urinary tract infections, ECA is an invariant carbohydrate restricted to *Enterobacterales* and is absent only in endosymbionts with significantly reduced genomes (Rai and Mitchell 2020; Jenkins et al. 2017; Kunin 1963; Kunin et al. 1962). ECA has been shown to play a role in the pathogenesis of several members within the order of *Enterobacterales* (Aguiniga et al. 2016; Kalynych et al. 2014; Gilbreath et al. 2012; Armbruster et al. 2017; Holmes et al. 2023; Guérin et al. 2020). For example, *Salmonella enterica* serovar Typhimurium LT2 strains lacking ECA display reduced virulence, heightened sensitivity to bile salts, and establish a low‐level persistent infection in mouse models (Kalynych et al. 2014; Gilbreath et al. 2012).

ECA is defined by recurring trisaccharide repeat units (RUs) of GlcNAc (*N*‐acetyl‐d‐glucosamine), ManNAcA (*N*‐acetyl‐d‐mannosaminuronic acid), and Fuc4NAc (4‐acetamido‐4,6‐dideoxy‐d‐galactose) (Männel and Mayer 1978). There are three forms of ECA, (i) ECA_LPS_, which replaces O‐antigen (Oag) on the LPS core; (ii) ECA_PG_, a cell‐surface form linked to diacylglycerol‐phosphate; and (iii) ECA_CYC_, a soluble, cyclic form found in the periplasm (Goździewicz et al. 2015; Kunin 1963; Männel and Mayer 1978; Kajimura et al. 2005). ECA_PG_ and ECA_LPS_ vary in the number of RUs with a range of 1 to 14 and has a modal value of 5 to 7 at 37°C (Mitchell et al. 2018; Barr et al. 1999), whereas ECA_CYC_ has a defined number of exactly 4 RUs in *Escherichia coli* K‐12 (Kajimura et al. 2005). Structural analysis studies including crystallography, nuclear magnetic resonance (NMR), and molecular dynamics indicate that ECA_CYC_ adopts either a rhomboidal or square conformation (Färnbäck et al. 2003; Erbel et al. 2004). We have previously found that ECA_CYC_ contributes to OM integrity in *E.coli* K‐12 and mediates resistance to detergents and bile salts (Mitchell et al. 2018).

ECA biosynthesis genes reside mainly in the *wec* operon and employs a pathway analogous to the Wzy/Wzz‐dependent pathway of Oag synthesis (Eade et al. 2021; Rick et al. 1985; Barr and Rick 1987; Barr et al. 1989, 1999; Bertani and Ruiz 2018; Erbel et al. 2003). After a single undecaprenyl‐phosphate‐linked ECA RU is synthesized, WzxE flips the intermediate (lipid III^ECA^) to the outer leaflet of the inner membrane (Liu et al. 1996; Rick et al. 2003; Rai and Mitchell 2020). WzyE and WzzE form a complex where the translocated undecaprenyl‐phosphate‐linked ECA subunits are polymerized by WzyE and the number of RUs in the resulting chain is regulated by WzzE (Leo, Tran, and Morona 2021; Maczuga, Tran, Qin, and Morona 2022; Nath and Morona 2015; Weckener et al. 2023). WzzE is an octameric inner‐membrane protein with a large periplasmic domain (Figure S1) (Tocilj et al. 2008; Kalynych, Yao, et al. 2012; Collins et al. 2017; Kalynych et al. 2015). A cryo‐EM structure from *Pectobacterium atrosepticum* suggest that WzyE resides within the central lumen formed by WzzE's transmembrane helices (Weckener et al. 2023).

WzzE is a member of the class 1 polysaccharide co‐polymerase (PCP1) family of chain length regulators, which includes among others, WzzB, FepE, and Wzz (Wiseman et al. 2021, 2023; Morona et al. 2009). Despite low sequence identity, PCP1 proteins share a conserved architecture consisting of two transmembrane helices and a bell‐shaped periplasmic domain (Tocilj et al. 2008; Kalynych, Yao, et al. 2012; Collins et al. 2017; Kalynych et al. 2015). In *Shigella flexneri* , WzzB C‐terminal transmembrane helix two (TMH2) is known to interact with WzyB, the polymerase, and is necessary for Oag chain‐length regulation (Ascari et al. 2022; Leo, Teh, et al. 2021). It has also been shown that WzzB and WzyB co‐purify in the absence of chemical crosslinkers, and that TMH2 of WzzE alone is also sufficient enough to maintain interaction with WzyB (Leo, Tran, and Morona 2021). Furthermore, a bidirectional interaction between Oag and ECA synthesis has been demonstrated, in which WzzE is capable of partially regulating Oag chain length through its interaction WzyB (Leo, Tran, and Morona 2021). It has also been shown that mutations disrupting Oag chain‐length regulation are found throughout Wzz and are not restricted to TMH2 (Whitfield et al. 2020). Studies of FepE have shown that mutant constructs containing single amino acid substitutions consistently produced LPS with shortened O‐antigen chains, without a consistent correlation between oligomer size and functional activity (Tran and Morona 2013). Furthermore, mutations in *fepE* were shown to significantly reduce the minimal inhibitory concentration (MIC) of *S* . Typhimurium for bile acids in vitro as *fepE* promotes the formation of very long Oag chains (Crawford et al. 2012).

WzzE plays a more complex role in ECA synthesis than other PCP1s do in Oag production. Loss of WzzE generates linear ECA with increased heterogeneity—producing ECA with both shorter and longer RUs—rather than the pronounced shortening typical of PCP1 deletions in Oag pathways (Leo, Tran, and Morona 2021; Ascari et al. 2022; Huszczynski et al. 2019; Kalynych et al. 2011). In contrast to linear ECA, strains lacking WzzE also fail to produce ECA_CYC_, indicating that WzzE is required for ECA_CYC_ synthesis despite a lack of known catalytic activity (Rai and Mitchell 2020; Mitchell et al. 2018; Kajimura et al. 2005). Additionally, WzzE plays a role in regulating levels of ECA_PG_ in *E. coli* K‐12. ElyC regulates production of ECA_PG_ in such a way that loss of ElyC causes large increases in ECA_PG_ production (Rai et al. 2021). This regulation is dependent on WzzE and likely on ECA_CYC_, and changes in ECA_PG_ levels with loss of ElyC are much less in the absence of WzzE (Mitchell et al. 2018; Rai et al. 2021). Finally, ECA takes on aberrant activity in the absence of the anterograde phospholipid transporter, YhdP, causing increased sensitivity to vancomycin and the detergent SDS (Mitchell et al. 2018). This phenotype is reversed when *wzzE* is absent.

Several non‐exclusive hypotheses for how PCPs regulate chain length of linear carbohydrates have been proposed (Islam and Lam 2014; Leo, Tran, and Morona 2021; Wiseman et al. 2021, 2023; Kintz and Goldberg 2011; Papadopoulos and Morona 2010; Morona et al. 1995; Weckener et al. 2023). (i) The inner or outer surface of the PCP may act as a molecular ruler for chain length regulation (Kintz and Goldberg 2011). Specifically, the PCP may bind the extending carbohydrate, facilitating catalysis until the polymer length breaks the interaction (Kalynych, Valvano, and Cygler 2012). (ii) The PCP could serve as a scaffold on which multiple polymerase molecules assemble (Tocilj et al. 2008). (iii) The elongating carbohydrate could extend in the lumen of the PCP until it reaches the top of the periplasmic domain and is exposed (Islam and Lam 2014). (iv) The PCP could interact with the polymerase to sequester short glycan chains from downstream ligases (i.e., WaaL), increasing the processivity of polymerization (Hong et al. 2023). Finally, (v) the loops at the top of the periplasmic bell may move in an alternating up and down arrangement which allows for the polysaccharide chain to extend using a “ratchet”‐type mechanism (Wiseman et al. 2023). However, the mechanism(s) used by WzzE for ECA_CYC_ synthesis is unknown. Here, we used site‐directed mutagenesis and biochemical analysis to test whether WzzE requires only polymerase‐interaction/chain‐length regulation to produce ECA_CYC_, or if an additional function of WzzE contributes to ECA_CYC_ biogenesis. Our findings demonstrate that polymerase interaction and chain‐length regulation are necessary for ECA_CYC_ biosynthesis. Moreover, we found that ECA_CYC_ synthesis genetically separated from linear ECA regulation, suggesting an additional function of WzzE in ECA_CYC_ synthesis.

## 2 Results

### 2.1 Plasmid‐Based *wzzE* Allows Analysis of *wzzE* Mutant Function

**FIGURE 1:**
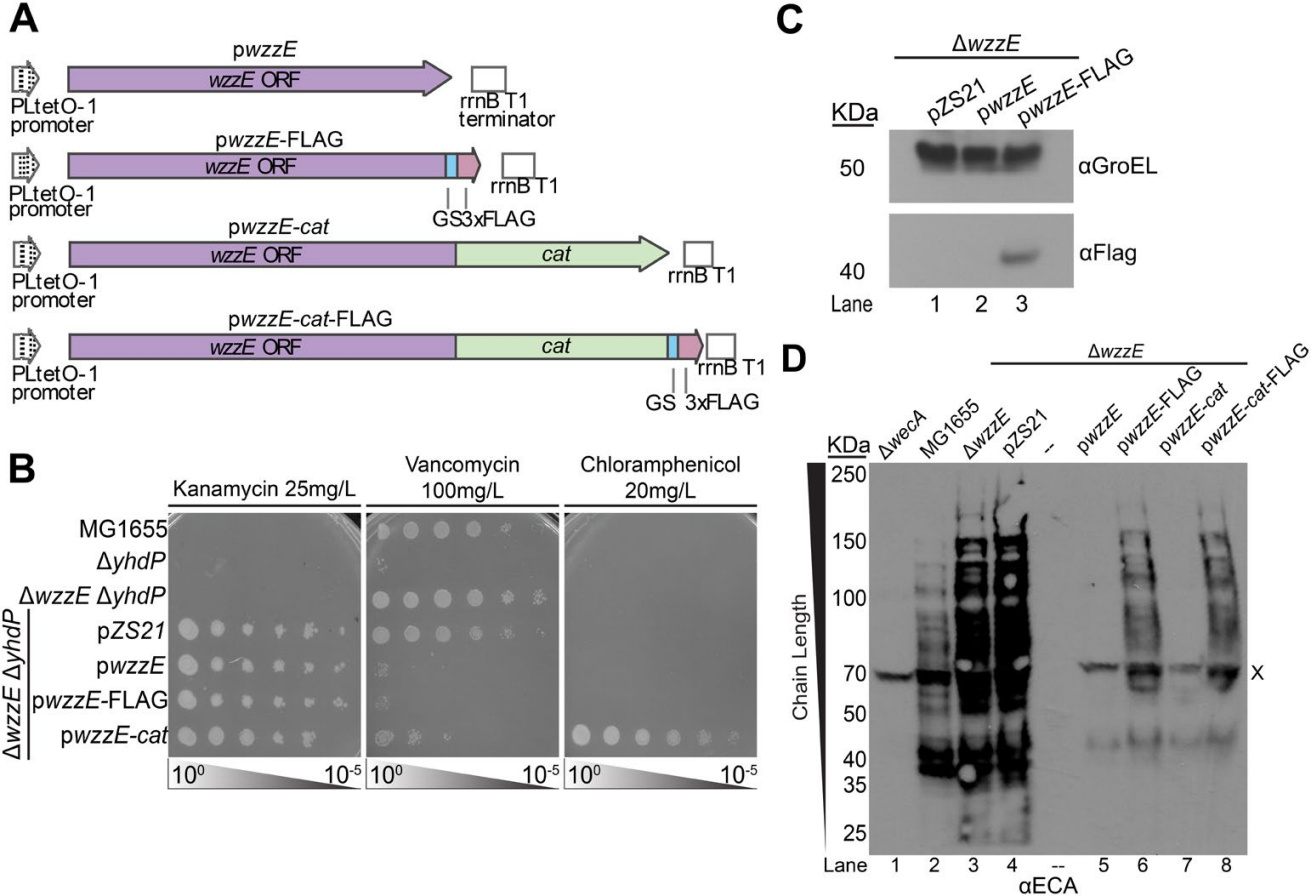
Plasmid‐based expression of *wzzE* partially complements a chromosomal *wzzE* deletion. (A) Diagrams of constructs for expression of wild‐type WzzE, WzzE with a C‐terminal glycine‐serine linker and triple FLAG tag, WzzE translationally fused with chloramphenicol acetyltransferase (Cat), and WzzE fused with Cat and triple FLAG are shown. The indicated constructs were expressed from the low copy number pZS21 plasmid (Lutz and Bujard 1997). (B) The vancomycin sensitivity of a Δ*yhdP* strain can be suppressed by loss of WzzE (Mitchell et al. 2018). When the Δ*wzzE* Δ*yhdP* strain carried the pZS21 empty plasmid, it remained resistant to vancomycin as assayed by EOP but became sensitive when *wzzE, wzzE*‐FLAG, or *wzzE*‐*cat* was expressed, indicating all three constructs complemented Δ*wzzE*. In addition, translational fusion of *cat* and *wzzE* conferred chloramphenicol resistance, suggesting this construct could readout WzzE stability and multimerization based on chloramphenicol resistance. (C) WzzE‐FLAG was detectable by immunoblotting. GroEL serves as a loading control. (D) ECA chain‐length regulation was assayed through immunoblotting using an ECA antibody. Plasmid‐based expression of WzzE restored chain‐length regulation of linear ECA, although lower levels of ECA and shorter chain‐length were observed than in the wild‐type strain for the p*wzzE* and p*wzzE* constructs, while the p*wzzE‐*FLAG and p*wzzE‐cat‐*FLAG constructs complemented better. The Δ*wecA* strain produces no ECA and serves as negative control. X: nonspecific band. Molecular weights are based on protein standards. All images are representative of at least three independent experiments.

To define the functions of WzzE required for ECA_CYC_ synthesis in *E. coli* K‐12, we examined the effects of *wzzE* mutations analogous to known residues affecting PCP1 Oag regulation. We first generated a set of *wzzE* constructs constitutively expressed by the *tet* promoter on the low copy number pZS21 plasmid (Lutz and Bujard 1997), which does not contain a Tet repressor (Figure 1A). These included wild‐type *wzzE* (p*wzzE*) and C‐terminal fusions bearing a glycine‐serine linker and triple FLAG tag (p*wzzE*‐FLAG), a translational fusion to chloramphenicol acetyltransferase (p*wzzE‐cat*), and a p*wzzE‐cat* triple FLAG tag fusion (p*wzzE‐cat‐*FLAG). Based on the topology of WzzE, we anticipated that the C‐terminal fusions would be exposed in the cytoplasm, thereby allowing Cat to function. Thus, these constructs allowed us to potentially identify defects in WzzE's folding or stability as measured by chloramphenicol resistance. This approach was informed by a translational coupling methodology previously used as a tool in *E. coli* (Mendez‐Perez et al. 2012; Sieber 2003), in which an unstable or degraded protein construct was shown to not allow chloramphenicol resistance. Additionally, we hypothesized that the formation of the functional cat trimer (Andreeva et al. 2000) is more likely to occur if WzzE is properly multimerized, allowing Cat monomers to come into contact (with up to two Cat trimers potentially formed per octamer), giving insight into potential disruptions in octamerization. This assay is particularly useful because the addition of the bulky Cat (26KDa) protein likely imposes stress on WzzE folding. However, the chloramphenicol resistance results remain suggestive due to the potential limitations of this assay, such as the multimerization of Cat affecting octamer formation (i.e., a loss of chloramphenicol resistance is more indicative of a loss of stability and/or multimerization than chloramphenicol resistance is indicative of multimerization).

We next assessed whether these constructs complemented Δ*wzzE*. In the absence of *yhdP*, ECA causes sensitivity to vancomycin in a *wzzE* dependent manner (Mitchell et al. 2018). Therefore, we expressed the *wzzE* constructs in a Δ*wzzE* Δ*yhdP* strain and tested for vancomycin sensitivity. Our results demonstrated each construct complemented Δ*wzzE* by restoring vancomycin sensitivity (Figure 1B). Additionally, p*wzzE*‐*cat* conferred chloramphenicol resistance to the strain, indicating that the Cat fusion was functional. To determine if the p*wzzE*‐FLAG construct could be detected, we immunoblotted for FLAG using GroEL as a loading control (Figure 1C) and determined that the fusion protein could be clearly detected.

To determine whether linear ECA regulation was restored in the *wzzE* plasmid constructs, we first performed immunoblots for linear ECA (Figure 1D) using a polyclonal ECA antibody (Leo, Tran, and Morona 2021). Our results revealed a range of ECA bands consistent with previous publications (Mitchell et al. 2018; Rai et al. 2021; Morris and Mitchell 2023), with increasing molecular weight indicating molecules with more RUs. The distribution of ECA chain lengths was wider in a Δ*wzzE* mutant (Lane 3) than in wild‐type cells (Lane 2) with an increase in longer and shorter ECA bands, indicating loss of chain‐length regulation. We also observed more linear ECA with Δ*wzzE* as expected from previous findings (Rai et al. 2021; Mitchell et al. 2018). For simplicity, we will refer to these changes as “loss of chain‐length regulation”. Expression of p*wzzE* (Lane 5) but not the empty vector (Lane 4) restored chain‐length regulation, albeit with a lower modal chain length than the wild‐type strain and much less linear ECA overall. These changes may suggest overactivity of WzzE due to multi‐copy expression. The same pattern was observed for the p*wzzE‐cat* construct (Lane 7); however, the p*wzzE*‐FLAG and p*wzzE‐cat‐*FLAG constructs resulted in chain‐length regulation closer to that of wild type. Overall, our results demonstrate plasmid‐based expression of WzzE is sufficient for efficiently screening loss‐of function phenotypes with respect to linear ECA regulation. However, because linear ECA is detected at relatively low levels in this assay, we determined that it was not suited for identifying more subtle effects on chain length regulation or linear ECA production. We, therefore, first screened mutations in the plasmid‐based system and then generated and analyzed chromosomal versions of key mutations to more clearly define the effects of these mutations on chain‐length regulation and linear ECA production.

### 2.2 LC/MS‐Based Analysis Allows Relative Quantification of ECA_CYC_ Abundance

**FIGURE 2:**
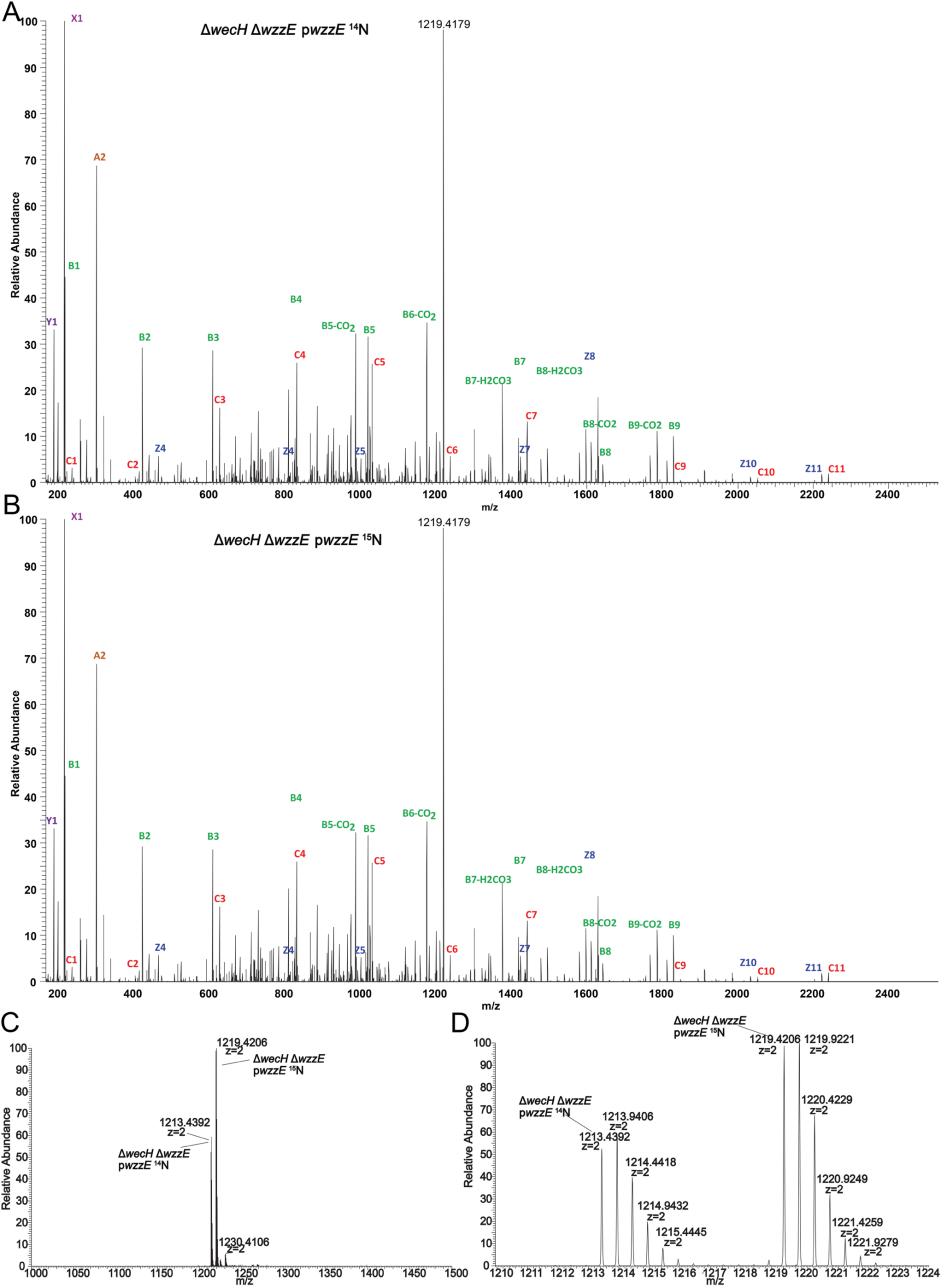
Structural confirmation of ECA_CYC_ using tandem mass spectrometry. (A) MS2 spectrum of the unlabeled ECA_CYC_ ion (m/z 1213.4) at collision energy 60 V. The series of ion fragments corresponding to the sequence of the ECA_CYC_ are labeled (blue) in the order of (GlcNAc‐Fuc4NAc‐ManNAc) with mass sequential m/z differences of 187 (x), 217 (*), and 203 (+), respectively. The source strain carried Δ*wecH* to prevent non‐stochiometic acetylation of ECA. (B) Cells were grown with a heavy nitrogen (^15^N) source overnight prior to ECA_CYC_ sample harvesting. MS2 spectrum of the ^15^N labeled ECA_CYC_ ion (m/z 1219.4) at collision energy 60 V. A similar patter of peaks is observed compared to (A) with a shift in m/z between A and B corresponding to the number of ^15^N in each ion (also see Dataset S1). (C) Complementation of Δ*wzzE* with p*wzzE* restores ECA_CYC_ production. Labeled and unlabeled samples were grown as in (B) and combined before purification of ECA_CYC_, allowing for relative quantitation of the ECA_CYC_ abundance in the samples. (D) Expanded view of the m/z 1210–1224 region from Δ*wecH* Δ*wzzE* p*wzzE* samples, resolving the uniform isotopic variants of the labeled and unlabeled ECA_CYC_.

Next, we tested whether the plasmid‐based *wzzE* constructs rescued ECA_CYC_ synthesis. We expressed p*wzzE* and empty vector pZS21 plasmid in the Δ*wzzE* Δ*wecH* strain. WecH non‐stoichiometrically acetylates ECA (Kajimura et al. 2006), and deleting *wecH* allows ECA_CYC_ to be quantitated as a single mass species (Kajimura et al. 2006; Mitchell et al. 2018). Immunoblot analysis of linear ECA species across these strains showed no detectable changes in linear ECA regulation with Δ*wecH* (Figure S2A), although there was a decrease in linear ECA levels with Δ*wecH* in both the wild‐type and Δ*wzzE* backgrounds. Unlike the two linear forms of ECA, ECA_CYC_ is uncharged and cannot be detected by immunoblotting. ECA_CYC_ has an invariable number of RUs (4) in *E. coli* K‐12 and exhibits a deprotonated ([M‐H]^−^) monoisotopic mass of 2427.889 Da ([M‐2H]^2−^ m/z 1213.436) in the absence of acetylation. Incorporation of heavy ^15^N results in a 12 Da mass increase due to labeling of the 12 nitrogen atoms present in ECA_CYC_ (one per sugar monomer) ([M‐2H]^2−^ m/z 1219.419) (Figure S2B). This labeling allows samples for ECA_CYC_ quantification to be mixed before purification, ensuring equal yield from the samples that are compared. This approach enables internal normalization within each sample and allows for accurate relative comparisons of ECA_CYC_ abundance based on the ratio of labeled to unlabeled species. To determine whether we could conclusively identify ECA_CYC_ in these samples, we grew cultures in ^15^N, mixed them with their corresponding unlabeled (^14^N) cultures prior to purification, and performed LC–MS/MS analysis, as shown in Figure 2A. The MS2 fragmentation of the doubly charged ion at m/z 1213.4 yielded detailed fragmentation patterns (Figure 2A, Dataset S1). Depending on the fragmentation initiation site, the spectra displayed a reproducible pattern characterized by stepwise fragmentation of individual sugar moieties within the ECA_CYC_ repeat unit (Figures 2A and S3). This fragmentation sequence confirms the arrangement of the sugar moieties (GlcNAc‐ManNAc‐Fuc4NAc) and the observed sequential m/z differences of 187, 203, and 217 respectively, thereby supporting the cyclic structure of ECA_CYC_ and the proposed composition and organization of the polymer. Notably, the labeled doubly charged ion at m/z 1219.4 exhibited a fragmentation pattern similar to that of the unlabeled ion (Figure 2B), with each peak shifted in m/z according to the number of nitrogen atoms present, ranging from 1 to 12 (Dataset S1).

We employed the same isotopic labeling strategy used in the LC–MS/MS experiments in our LC–MS quantification experiments. When labeled and unlabeled samples carrying p*wzzE* were combined, we detected peaks at the same retention time of both the unlabeled and labeled m/z (Figures 2C,D and S2C). The m/z peaks match within 5 ppm of the simulated spectra for the labeled and unlabeled forms of ECA_CYC_ (Figure S2C). We also demonstrated that the LC peak containing ECA_CYC_ could only be detected in the Δ*wzzE* Δ*wecH* strain carrying p*wzzE* and not the empty vector (Figure S2D). Furthermore, ECA_CYC_ production from the p*wzzE* and p*wzzE‐cat* constructs was very similar (Figure S2E). These data confirm that plasmid‐based expression of *wzzE* can facilitate the synthesis of ECA_CYC_, making this an appropriate system to investigate the role of WzzE in ECA_CYC_ biosynthesis.

### 2.3 Conserved Glycine's in TMH2 Are Necessary for WzzE Function

After validating our WzzE functional assays, we next examined how TMH2 contributes to WzzE's function. TMH2 has been shown to mediate interactions between WzzB and Wzy in O‐antigen synthesis (Leo, Tran, and Morona 2021), and prior mutagenesis studies of WzzB identified a conserved GXXXG motif in the TMH2 region of WzzB from *Shigella flexneri Y* that is important for function (Daniels and Morona 1999), likely due to effects on helix–helix packing (Teese and Langosch 2015; Prakash et al. 2011; Mueller et al. 2014). A corresponding double mutant in *Shigella flexneri Y* (G305A/G311A WzzB; G306A/G312A in *E. coli* ) disrupts Oag chain length regulation (Papadopoulos et al. 2016); therefore, we hypothesized that the analogous mutations to the GXXXG motif located in WzzE would play a significant role in mediating the helix–helix packing of the transmembrane region and its interaction with WzyE.

**FIGURE 3:**
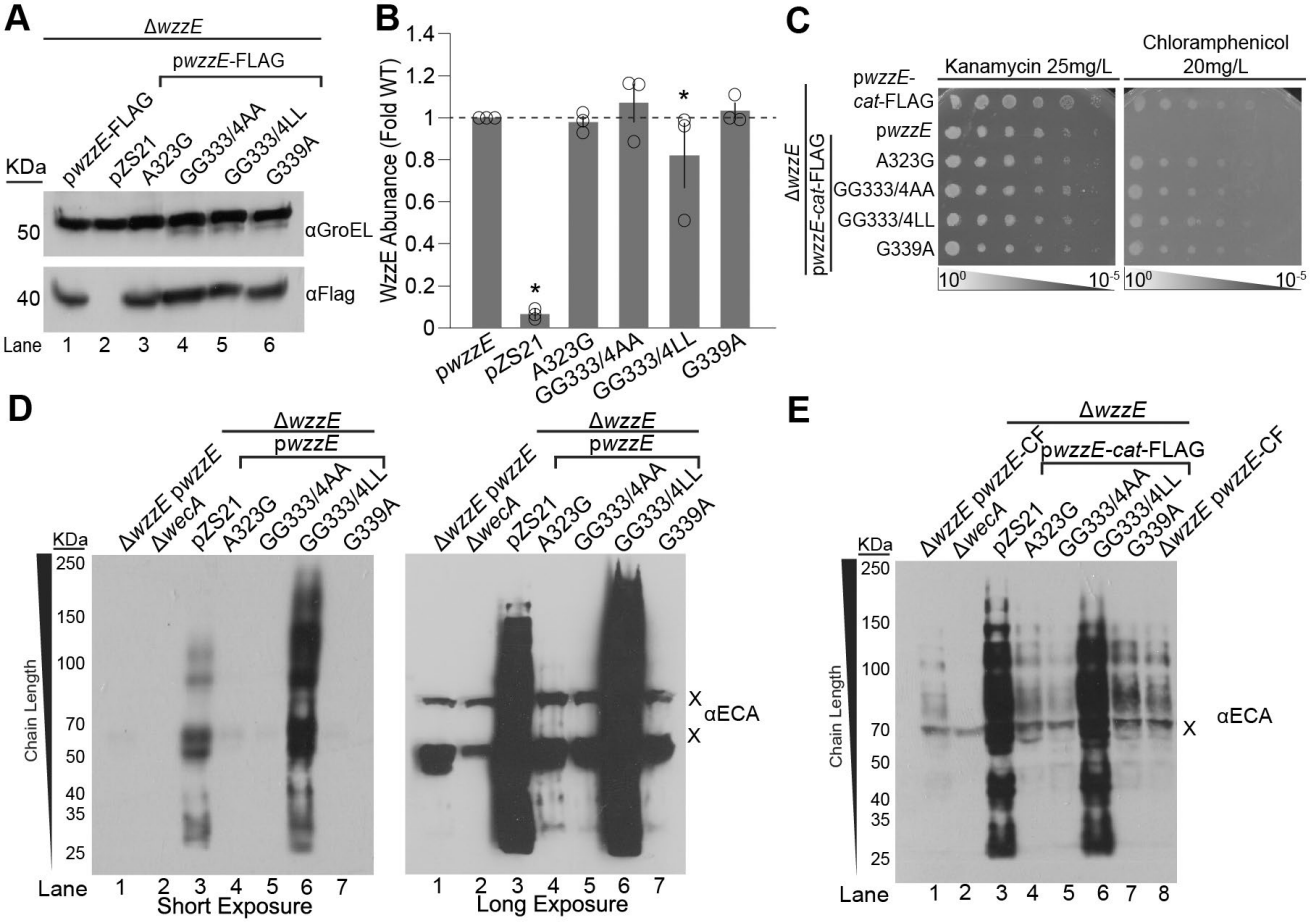
Alteration of WzzE TMH2 can prevent ECA chain length regulation without affecting WzzE stability. (A) The effect of the indicated *wzzE* site‐directed mutations on WzzE‐FLAG protein levels was assessed using immunoblotting. GroEL serves as a loading control. (B) Densitometry was used for quantification of the immunoblots in (A). Levels of WzzE‐FLAG compared to GroEL are shown normalized to the wild‐type *wzzE* complemented strain. All variants show similar protein levels to wild‐type WzzE, except for a small, but significant decrease in the WzzE^GG333/4LL^ variant. Data are shown as the average of three biological replicates ± SEM and individual datapoints. *p < 0.05 by Mann–Whitney test. (C) Strains carrying the indicated plasmids were assayed for their chloramphenicol resistance by EOP as a proxy for WzzE stability and multimerization ability. All *wzzE* mutants retained full chloramphenicol resistance, suggesting WzzE stability and multimerization. (D) Chain‐length regulation of the linear ECA forms by the indicated WzzE variants was assayed by immunoblotting. Short and long exposures are shown. “X” indicates non‐specific bands. WzzE^GG333/4LL^ showed loss of chain‐length regulation while the other variants retained wild‐type function. The Δ*wecA* strain serves as a negative control. (E) Chain‐length regulation of the WzzE‐Cat‐FLAG variants was assayed by immunoblot. Similar phenotypes were observed to those found in the WzzE variants. “X” indicates non‐specific band. Molecular weights are based on protein standards. All images are representative of at least three independent experiments.

To test this, we generated TMH2 mutations (Figure S1B) in the p*wzzE* constructs described in Figure 1A. We first sought to determine whether the levels of WzzE protein produced by p*wzzE‐*FLAG or p*wzzE‐cat‐*FLAG were altered by the mutations (Figures 3A,B and S4A). Our results showed a quite small but statistically significant decrease in protein abundance for our WzzE^GG333/4LL^ in the WzzE‐FLAG fusion (Figure 3A,B), whereas no decrease is apparent in the WzzE‐Cat‐FLAG fusion (Figure S4A). As the levels of the WzzE^GG333/4LL^ variant are 82% of wild type on average and as high as 98% in individual replicates, the biological relevance of this difference is unclear. To determine whether the mutations might have caused alterations to protein stability or multimerization, we utilized our p*wzzE‐cat* construct to assay chloramphenicol resistance conferred by the fusion protein. None of our mutations showed any chloramphenicol sensitivity (Figures 3C and S4B), suggesting that they are largely stable and multimerized, despite the small decrease in abundance for WzzE^GG333/4LL^. Next, we set out to determine if the mutations affected WzzE's ability to regulate ECA chain length by performing ECA immunoblots. Three of the TMH2 mutations, WzzE^A323G^, WzzE^GG333/4AA^, and WzzE^G339A^ (Lanes 4, 5, and 7) retained WzzE function (Figure 3D,E). In contrast, WzzE^GG333/4LL^ (Lane 6) markedly disrupted chain‐length regulation, resembling or exceeding the defect observed in our empty‐vector control (Lane 3). These results identify the TMH2 GXXXG region as a key determinant of WzzE‐mediated ECA chain‐length regulation.

### 2.4 The TMH2 GXXXG Motif Affects ECA_CYC_ Synthesis

**FIGURE 4:**
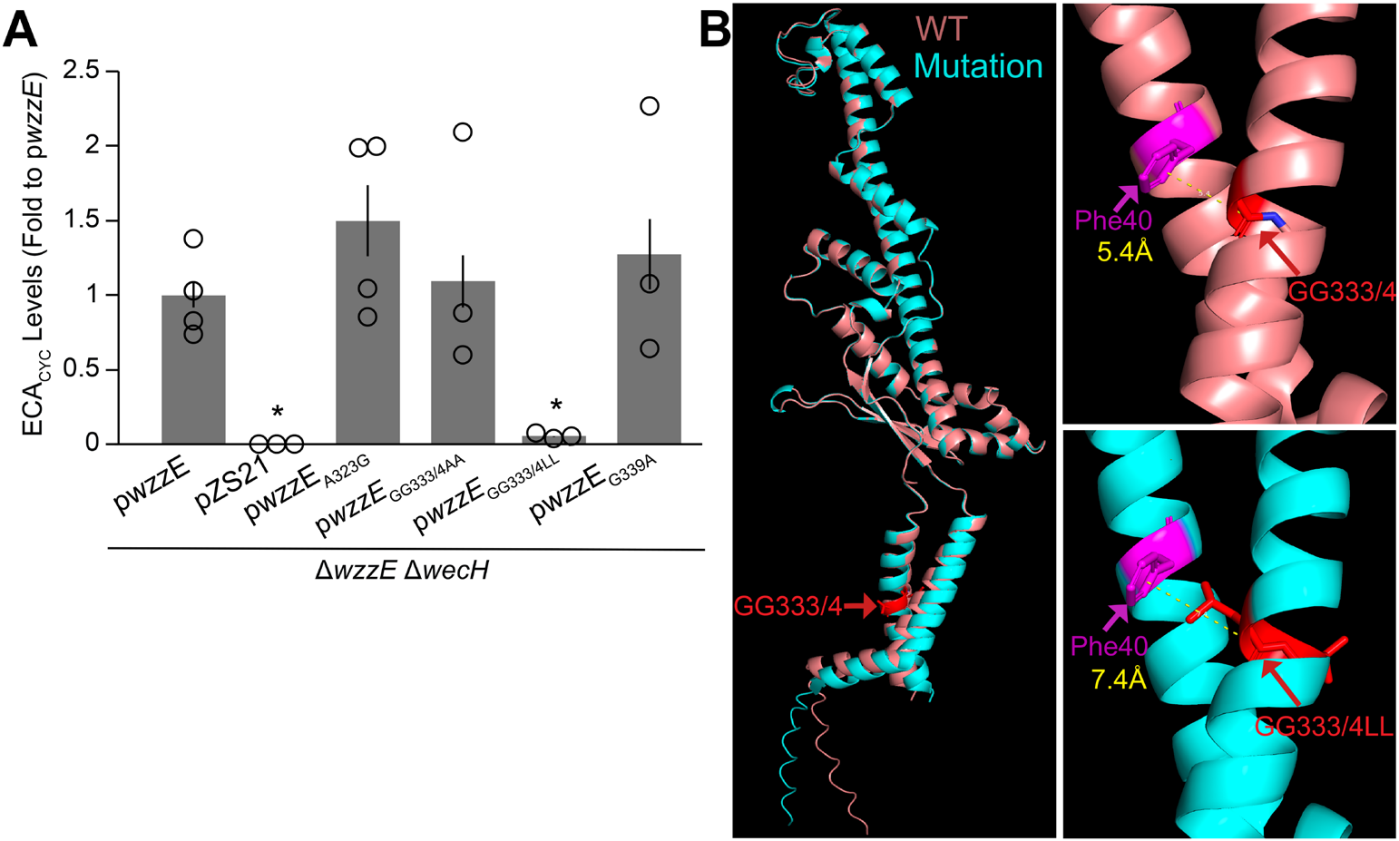
Conserved glycine residues in WzzE TMH 2 are required for ECA_CYC_ synthesis. (A) The strain carrying a plasmid with wild‐type *wzzE* was grown with ^15^N to label ECA and then combined with an unlabeled strain carrying the indicated *wzzE* mutant or control. ECA_CYC_ was purified and the relative amount of unlabeled to labeled ECA_CYC_ was determined by LC–MS. WzzE^GG333/4LL^ showed a loss of the ability to produce ECA_CYC_. All plasmids were expressed in a Δ*wzzE* Δ*wecH* strain background to prevent non‐stochiometric acetylation of ECA. Data are shown as the average of three to four biological replicates ± SEM and as individual datapoints. *p < 0.05 by Mann–Whitney test. (B) The predicted structure of a WzzE octamer with WzyE was calculated using AlphaFold 3 (Abramson et al. 2024). The structures of wild‐type WzzE and WzzE^GG333/4LL^ monomers were aligned with the mutated residues shown in red. The insets show the predicted distance between the two WzzE TMH in the wild type WzzE and WzzE^GG333/4LL^.

Given the strong defect of WzzE^GG333/4LL^ in regulating ECA chain length, we assessed whether the TMH2 mutations impair ECA_CYC_ biogenesis. We utilized LC–MS to detect and quantitate ECA_CYC_ in purified samples as in Figure 2C,D. We expressed the TMH2 mutants or controls in a Δ*wzzE* Δ*wecH* strain background and labeled the wild type p*wzzE* strain with ^15^N, while the mutants were grown with ^14^N. The quantification demonstrated that WzzE^A323G^, WzzE^GG333/4AA^, and WzzE^G339A^ did not significantly change ECA_CYC_ levels, whereas the WzzE^GG333/4LL^ mutant produced significantly reduced levels of ECA_CYC_, similar to those of the empty vector control (Figure 4A). These data demonstrate that the TMH2‐dependent chain‐length regulation is required for ECA_CYC_ synthesis.

To visualize how these mutations might alter WzzE function, we modeled the WzyE:WzzE(8) complex for wild‐type WzzE and WzzE^GG333/4LL^ using AlphaFold 3 (Abramson et al. 2024) (Figures 4B and S5; Table S1). Alignment of monomers revealed a ~2 Å expansion between TMH1 and TMH2 in WzzE^GG333/4LL^ consistent with steric disruption of helix–helix packing (Figure 4B). This structural shift provides a potential mechanistic basis for the loss of both chain‐length regulation and ECA_CYC_ biosynthesis in our GXXXG mutant.

### 2.5 The Periplasmic Domain of WzzE Functions in Linear ECA Regulation

After assessing the importance of WzzE TMH2 for ECA_CYC_ biosynthesis, we next sought to determine whether chain‐length regulating activities within the periplasmic domain of WzzE are also required for ECA_CYC_ biosynthesis. Twenty‐one periplasmic residues are conserved among WzzE, FepE, and WzzB in *E. coli* , suggesting that they are important for PCP1 function (Tocilj et al. 2008). We also hypothesized that non‐conserved residues could contribute to WzzE‐mediated chain‐length regulation or ECA_CYC_ synthesis. Therefore, we made mutations in a conserved cluster of predominantly hydrophobic residues located in the core of the ⍺/β domains—spanning strands β1 and β4, helix ⍺2, and the N terminus of helix ⍺6 (see Figure S1B). Additionally, we targeted non‐conserved residues throughout WzzE in positions similar to those that conferred changes to Oag chain length in other PCP1s (Tocilj et al. 2008).

**FIGURE 5:**
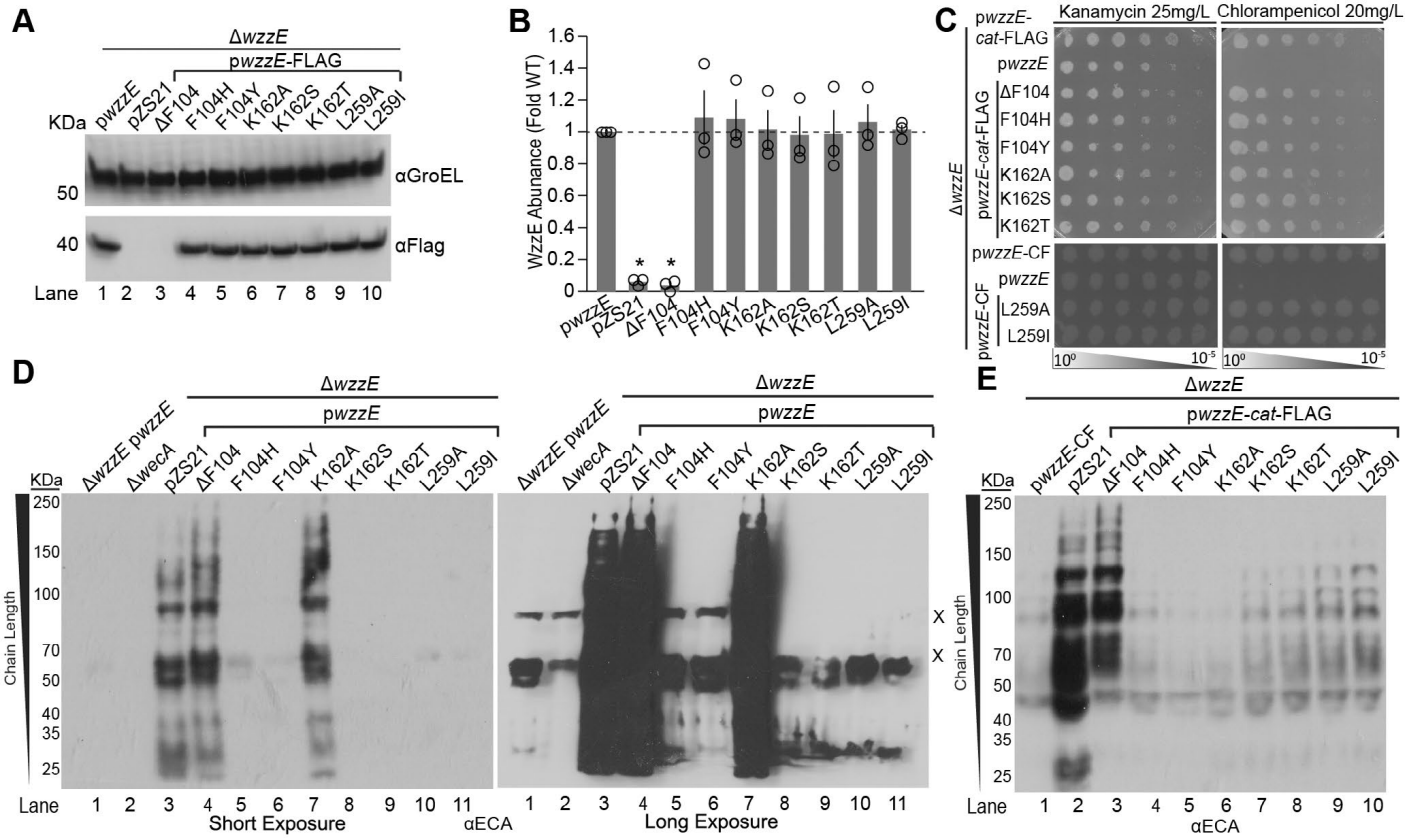
The periplasmic region of WzzE contributes to WzzE stability and chain length regulation. (A) Immunoblotting was used to determine protein levels for the indicated WzzE‐FLAG variants. GroEL serves as a loading control. (B) WzzE‐FLAG levels from (A) were quantified by densitometry. WzzE‐FLAG levels compared to GroEL are shown normalized to the wild‐type *wzzE* complemented strain. All variants are similar to wild type WzzE except for WzzE^ΔF104^ which shows significantly reduced protein levels. Data are shown as the average of three biological replicates ± SEM and individual datapoints. *p < 0.05 by Mann–Whitney test. (C) The indicated strains were assayed for WzzE‐Cat‐FLAG stability as measured by chloramphenicol resistance. All *wzzE* point mutations showed chloramphenicol resistance indicating stability and multimerization, with the exception of WzzE^ΔF104^ where the tag is cleaved (Figure S4C). (D) Linear ECA chain‐length regulation by WzzE variants was assayed by immunoblotting with ECA antibody. Long and short exposures are shown. The periplasmic point mutants have variable effects on chain length regulation as well as ECA_CYC_ biogenesis. The WzzE^ΔF104^ and WzzE^K162A^ variants showed loss of ECA chain‐length regulation while the other variants had wild‐type chain‐length regulation. The Δ*wecA* strain serves as negative control. (E) The effect of WzzE‐Cat‐FLAG variants on ECA chain‐length regulation was assayed by immunoblot. The variants show similar phenotypes as in (D) except for WzzE^K162A^, which shows normal chain‐length regulation and WzzE^ΔF104^ which shows partial chain‐length dysregulation. Molecular weights are based on protein standards. All images are representative of at least three independent experiments.

We first characterized the periplasmic mutants (Figure S1B) by examining WzzE protein levels using the p*wzzE*‐FLAG construct. WzzE^ΔF104^ (an in‐frame, single codon deletion) had undetectable WzzE‐FLAG protein levels, whereas all other mutants retained wild‐type protein accumulation (Figure 5A,B). When we examined the levels of WzzE‐Cat‐FLAG for WzzE^ΔF104^, we observed a faint band at the expected molecular weight of the fusion and a second, lower‐molecular‐weight band, indicating cleavage of the C‐terminal tag (Figure S4C). Given the cleavage of the tags, no conclusion could be drawn for the stability of WzzE^ΔF104^. All other protein levels were similar to those of wild type.

We next evaluated whether the *wzzE* mutations affect protein stability and/or multimerization by measuring chloramphenicol resistance conferred by the p*wzzE*‐cat fusion. WzzE^K162A^, WzzE^L259A^, and WzzE^F104Y^ caused partial or full chloramphenicol sensitivity in the p*wzzE*‐Cat fusion (Figure S4D) but showed full chloramphenicol resistance in the p*wzzE*‐Cat‐FLAG fusion, which better complements linear chain length regulation (Figure 5D). These differences suggest that these variants may exhibit mild instability or reduced multimerization.

All other mutants conferred chloramphenicol resistance similar to that of wild type in both fusions (Figures 5C and S4D). WzzE^ΔF104^ showed wild‐type levels of chloramphenicol resistance, likely reflecting cleavage of the C‐terminal tag. We also tested an additional 16 periplasmic‐domain mutations that caused chloramphenicol sensitivity in the WzzE‐Cat background, emphasizing the importance of this domain for WzzE structural stability and multimerization (Figure S6).

After characterizing the effect of the periplasmic mutations on WzzE levels and stability, we next examined how these periplasmic mutations affected ECA chain‐length regulation (Figure 5D,E). Our results show that WzzE^ΔF104^ and WzzE^K162A^ (lanes 4 and 7) disrupted WzzE function when expressed from p*wzzE* (Figure 5D). However, when expressed from p*wzzE*‐cat‐Flag, WzzE^ΔF104^ exhibited partial impairment of WzzE, while WzzE^K162A^ showed clear WzzE function (Figure 5E). These differences parallel the chloramphenicol resistance phenotypes observed for the corresponding fusions (Figures 5C and S4D). In contrast, WzzE^F104Y^, WzzE^F104H^, WzzE^K162S^, WzzE^K162T^, WzzE^L259A^, and WzzE^L259I^ (lanes 5, 6, 8–11) demonstrated WzzE function in both the p*wzzE* and p*wzzE‐cat‐FLAG* constructs. Together, these mutants provide a useful range of phenotypes for dissecting the requirements for ECA_CYC_ synthesis.

### 2.6 Periplasmic Residues of WzzE Are Important for ECA_CYC_ Biogenesis

**FIGURE 6:**
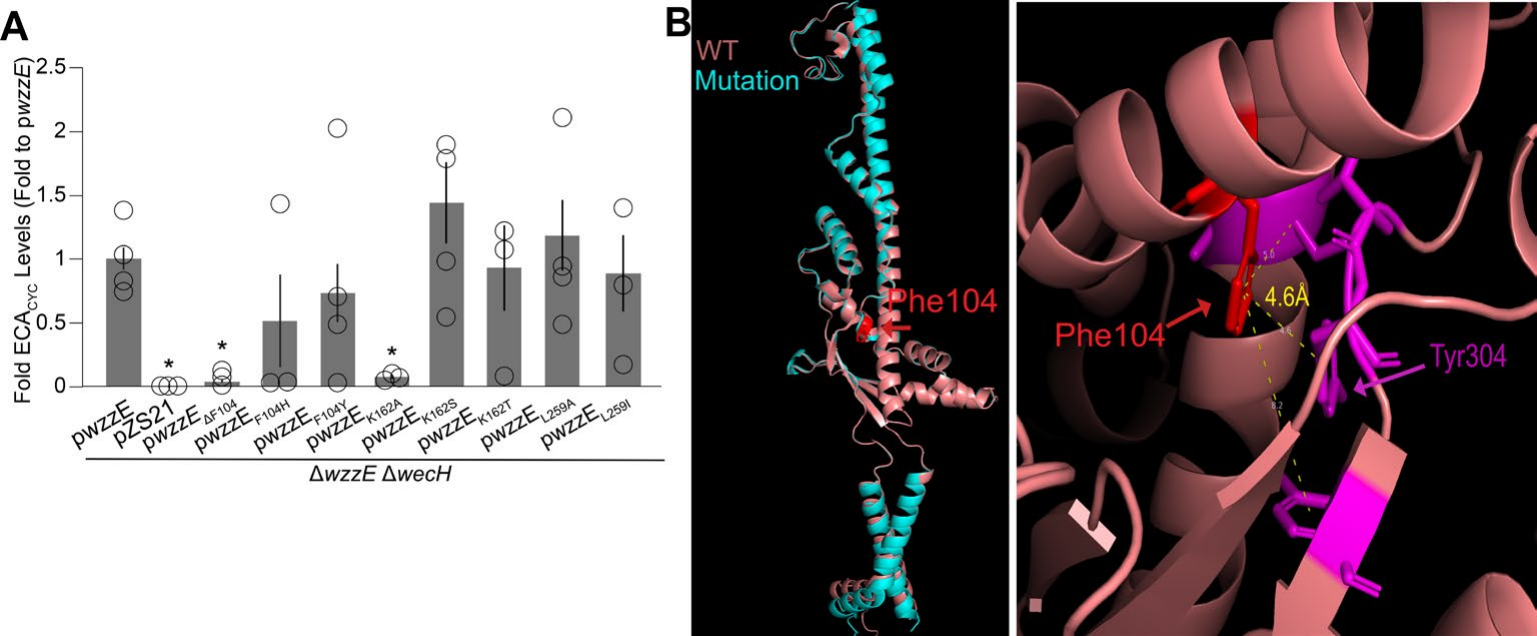
The periplasmic domain of WzzE contributes to ECA_CYC_ synthesis. (A) The strain carrying wild‐type *wzzE* was grown with ^15^N to label ECA and then combined with an unlabeled strain carrying the indicated *wzzE* mutant or control strain. After ECA_CYC_ purification, the relative amount of unlabeled to labeled ECA_CYC_ was determined by LC–MS. WzzE^ΔF104^ and WzzE^K162A^ had little to no ECA_CYC_ production while WzzE^F104H^ showed a variable phenotype. All strains included Δ*wecH* to prevent non‐stochiometric acetylation of ECA. Data are shown as the average of three to four biological replicates ± SEM and as individual datapoints. *p < 0.05 by Mann–Whitney test. (B) Structures predicted as in Figure 3 for wild‐type WzzE and WzzE^ΔF104^ were aligned. The mutated residue is shown in red on a WzzE monomer (B). The inset shows the interaction between alpha helix 2 and an adjoining loop in wild‐type WzzE.

We next assessed the impact of our periplasmic mutants on ECA_CYC_ biosynthesis by quantifying ECA_CYC_ levels using LC–MS. Both the WzzE^ΔF104^ and WzzE^K162A^ mutants exhibited impaired linear chain‐length regulation and produced little to no ECA_CYC_ (Figure 6A). This loss of ECA_CYC_ production could result from either (i) insufficient protein accumulation and/or stability or (ii) disruption of chain‐length‐regulating activities in these mutants. In contrast, WzzE^F104Y^, WzzE^K162S^, WzzE^K162T^, WzzE^L259A^, and WzzE^L259I^ produced ECA_CYC_ at levels comparable to wild type. WzzE^F104H^ showed highly variable ECA_CYC_ levels despite maintaining wild type chain‐length regulation and protein levels, suggesting that this mutation may have a specific ECA_CYC_ synthesis effect. These data highlight the importance of WzzE's periplasmic domain on ECA_CYC_ synthesis, likely through its structural role, its chain‐length‐regulating activity, and possibly a function specific to ECA_CYC_ synthesis. Given the differing effects of the F104 mutations on ECA_CYC_ synthesis and linear ECA chain‐length regulation, we examined their potential structural consequences using AlphaFold 3 (Abramson et al. 2024) and aligned wild type and WzzE^ΔF104^ complexes (Figures 6B and S5; Table S1). These models predict that F104 forms contacts with a neighboring loop, and alterations in these interactions may underlie the functional differences observed for substitutions at this residue.

### 2.7 ECA_CYC_ Biogenesis Is Genetically Separable From Chain‐Length Regulation

**FIGURE 7:**
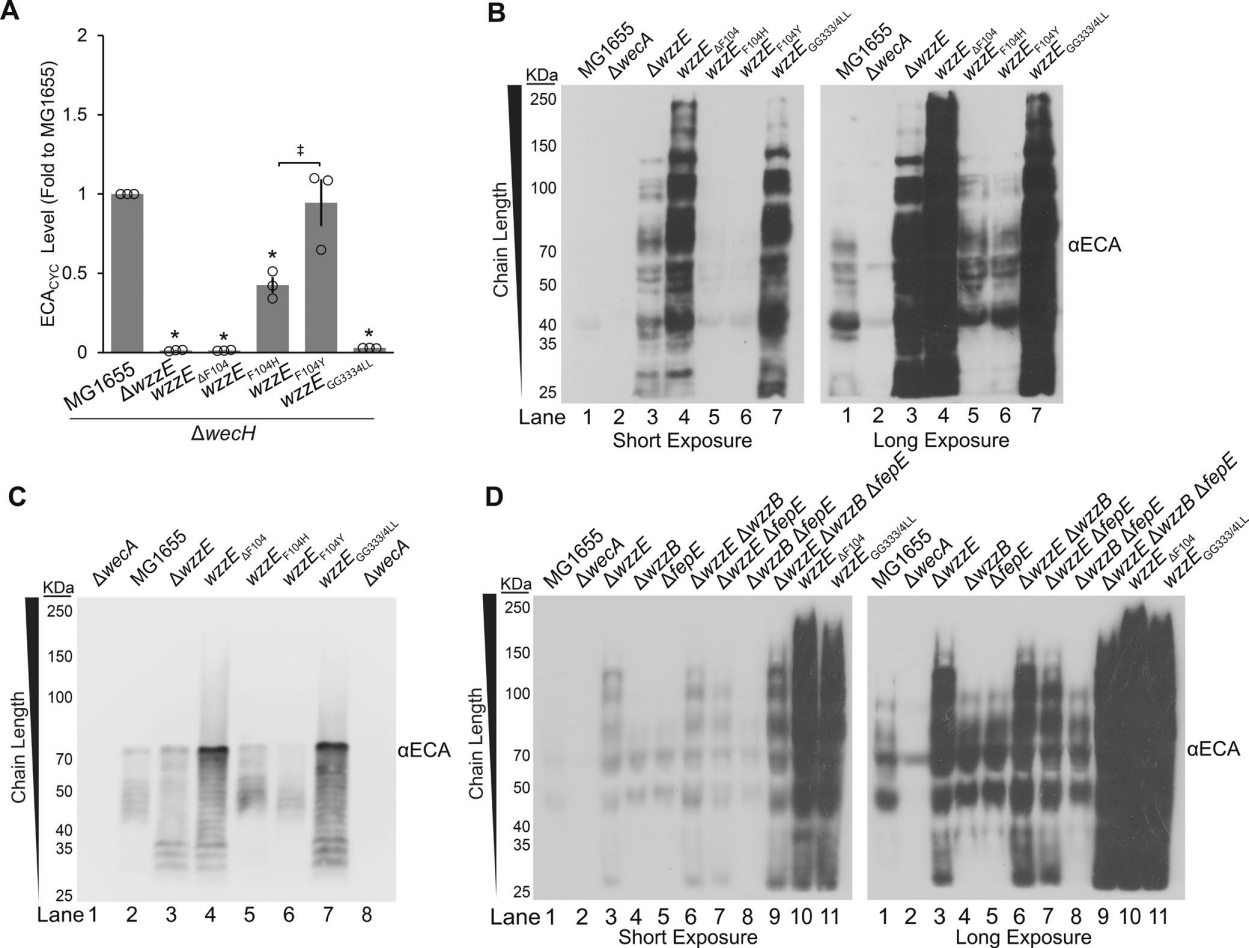
Levels of ECA_CYC_ production and chain‐length regulation are genetically separable. CRISPR‐Cas9 was used to make the indicated mutations to *wzzE* in its native locus. All strains contain *metE*::Tn*10*. (A) The strain wild type for *wzzE* was grown with ^15^N to label ECA and then combined with an unlabeled strain with the indicated *wzzE* mutant or control strain. After ECA_CYC_ purification, the ratio of unlabeled to labeled ECA_CYC_ was determined by LC–MS. All variants other than WzzE^F104Y^ produced significantly reduced ECA_CYC_ levels. Data are shown as the average of three biological replicates ± SEM and as individual datapoints. *p < 0.05 vs. wild type by Mann–Whitney test; ^‡^ *p* < 0.05 between the indicated strains by Mann–Whitney test. (B) Immunoblotting for ECA was conducted on the indicated chromosomal mutants. A short and long exposure are shown. The WzzE^ΔF104^ and WzzE^GG333/4LL^ variants resulted in linear ECA dysregulation, while the other mutations more closely resembled wild‐type linear ECA regulation. The Δ*wecA* strain serves as negative control. (C). ECA Immunoblotting was performed on the indicated chromosomal mutants after ProK treatment to more clearly assess chain length. WzzE mutants ΔF104 and GG333/4LL resulted in dysregulation of linear ECA, whereas F104Y and F104H strongly resembled wild‐type linear ECA regulation. (D) Immunoblotting was used to assay ECA chain‐length regulation in the indicated strains. Deletion of *wzzB* and/or *fepE* in combination with Δ*wzzE* did not cause the increased levels of linear ECA observed with the loss‐of‐function point mutants; however, the Δ*wzzE* Δ*wzzB* Δ*fepE* strain did have a small increase in linear ECA levels compared to Δ*wzzE* alone. Molecular weights are based on protein standards. All images are representative of at least three independent experiments.

Our plasmid‐based expression analyses of *wzzE* identified several point mutations with distinct effects on ECA_CYC_ production. Notably, substitution of the TMH2 residues (WzzE^GG333/4LL^) abolished chain‐length regulation and ECA_CYC_ synthesis, while maintaining protein stability and near‐wild‐type protein accumulation. Additionally, several mutations to residue F104 also produced variable effects on WzzE accumulation, chain‐length regulation, and ECA_CYC_ production. However, plasmid‐based expression of wild‐type *wzzE* yielded lower levels of linear ECA and reduced modal chain‐length compared with expression of *wzzE* from its native locus (Figure 1D), a difference likely contributing to some of the phenotypes observed in the plasmid system. We also noted substantial variability in ECA_CYC_ levels among periplasmic mutants, which we attributed to non‐native gene expression. To circumvent these limitations, we used CRISPR‐Cas9 to introduce select point mutations into the native *wzzE* locus and assayed their effect on linear ECA and ECA_CYC_. LC–MS analysis of purified ECA_CYC_ revealed that the chromosomal WzzE^ΔF104^ and WzzE^GG333/4LL^ variants produced little to no ECA_CYC_, similar to what is observed with Δ*wzzE* (Figure 7A). In contrast, WzzE^F104H^ produced measurable levels of ECA_CYC_ that were about 2‐fold lower than the wild type. WzzE^F104Y^ showed no significant change in ECA_CYC_ levels relative to wild‐type but was significantly different from WzzE^F104H^. Consistent with prior observations in our plasmid‐based expression system, WzzE^ΔF104^ and WzzE^GG333/4LL^ failed to regulate linear ECA (Figure 7B). Notably, WzzE^F104H^ and WzzE^F104Y^ displayed an identical chain‐length pattern (Figure 7B). The effects of all chromosomal mutants on linear ECA regulation were consistent in a Δ*wecH* strain (Figure S7A). We also noticed that the magnitude of chain‐length dysregulation in WzzE^ΔF104^ and WzzE^GG333/4LL^ exceeded that of Δ*wzzE*, and this could not be attributed to normalization (Figure S7B). This suggests that the presence of non‐functional WzzE may disrupt ECA synthesis differently than its complete absence.

The immunoblot conditions we generally use for ECA are optimized to minimize ECA degradation, as ECA can be delipidated by mild acid hydrolysis (Figure S8) (Basu et al. 1987). Under these conditions, residual protein in samples can cause ECA signal broadening, obscuring subtle changes in ECA distribution. Therefore, we examined linear ECA regulation for the chromosomal mutants in proteinase K (ProK) treated samples. WzzE^F104H^ and WzzE^F104Y^ still showed similar ECA chain length regulation, while WzzE^ΔF104^ and WzzE^GG333/4LL^ continued to show greater ECA dysregulation than ΔwzzE (Figure 7C). To identify any subtle differences in chain length between the WzzE^F104H^ and WzzE^F104Y^ variants, we quantified the ECA signal in the lower, middle, and upper molecular weight regions of the blot. There were no statistically significant differences between WzzE^F104H^ and WzzE^F104Y^, indicating that the observed change in ECA_CYC_ levels does not reflect a change in WzzE's ability to regulate ECA chain length (Figure S9).

Finally, we were surprised that the dysregulation of ECA chain length, and even more the levels of ECA, in the WzzE^ΔF104^ and WzzE^GG333/4LL^ mutants was greater than that of the Δ*wzzE* control. So, we wondered whether, in a Δ*wzzE* strain, the remaining PCP1s could partially regulate ECA chain length through interaction with WzyE. In this case, nonfunctional WzzE mutants would sequester WzyE from the other PCP1s, exacerbating dysregulation. Therefore, we assayed whether we could cause further disruption of ECA chain‐length regulation in a Δ*wzzE* strain by deleting *wzzB* and/or *fepE*. Deletion of Δ*wzzB*, Δ*fepE*, or both had no detectable effect on chain regulation in a Δ*wzzE* strain (Figure 7D), though the triple mutant showed a modest increase in linear ECA levels over those of the Δ*wzzE* strain. Overall, the identical regulation of linear ECA chain‐length by WzzE^F104H^ and WzzE^F104Y^ but differing effects on ECA_CYC_ synthesis demonstrate that substitutions at residue F104 differentially affect ECA_CYC_ abundance while maintaining linear ECA regulation. These findings suggest that the level of ECA_CYC_ synthesis and linear ECA chain‐length regulation are genetically separable.

## 3 Discussion

In this study, we constructed and characterized a set of *wzzE* site‐directed mutants to define which functions of WzzE are required for ECA_CYC_ biogenesis. Although previous mutagenesis studies have identified regions of WzzB in *Shigella flexneri* that are important for activity (Nath and Morona 2015; Ascari et al. 2022), no prior work has examined how WzzE contributes to the synthesis of ECA_CYC_. Here, we show that substitutions at F104 within the periplasmic domain and the GXXXG motif in TMH2 can compromise both ECA chain‐length regulation and ECA_CYC_ synthesis. Moreover, our data demonstrate that regulation of linear ECA chain length and the level of ECA_CYC_ production is genetically separable, as two substitutions at residue F104 exhibit divergent effects on ECA_CYC_ abundance while maintaining identical regulation of linear ECA chain length. Specifically, F104H showed a statistically significant reduction in ECA_CYC_ levels, whereas F104Y had near wild type abundance, despite both F104H and F104Y producing indistinguishable linear ECA banding patterns in immunoblot analyses. These observations indicate that WzzE‐mediated control of ECA chain length is necessary for ECA_CYC_ synthesis but is not sufficient to ensure proper ECA_CYC_ synthesis. Additionally, these findings highlight that specific residues within WzzE can differentially modulate outputs of WzzE, providing evidence that the mechanisms governing linear ECA chain‐length regulation and cyclic ECA synthesis are separable at the genetic level.

Despite low sequence conservation among PCP1s, their overall structures are highly similar, consisting of two transmembrane domains and an ⍺‐helix/β‐sheet domain within the periplasmic space that extends ~100 Å above the membrane (Tocilj et al. 2008; Kalynych, Yao, et al. 2012; Collins et al. 2017; Kalynych et al. 2015). PCP1s also harbor a conserved GXXXG motif that has been shown to contribute to carbohydrate chain‐length regulation. In *Shigella flexneri* , a G305A/G311A double mutant of *wzzB* that encompassed and extended outside of this motif resulted in short‐chain O‐antigen (Oag) (Daniels and Morona 1999; Papadopoulos et al. 2016), whereas single, double, and triple glycine to alanine mutations restricted to the GXXXG motif did not affect chain‐length (Collins et al. 2017; Daniels and Morona 1999). Motivated by these findings, we focused on residues within and around the GXXXG motif, characterized their effect on chain‐length regulation and ECA_CYC_ biogenesis, and identified two adjacent, conserved glycine residues in the GXXXG motif of WzzE—analogous to G305/G306 in WzzB—as critical for ECA chain‐length regulation and ECA_CYC_ biosynthesis. Interestingly, Ala substitutions at these positions had no detectable effect on chain‐length regulation or ECA_CYC_ production, whereas introduction of adjacent Leu residues led to pronounced dysregulation of ECA chain‐length and significantly reduced levels of ECA_CYC_.

GXXXG motifs often mediate dimerization and multimerization of transmembrane domains (Parrish et al. 2015; Faingold et al. 2012), suggesting that the motif may facilitate positioning of TMH1 and TMH2 of WzzE. In support of this idea, our structural modeling suggests increased distance between TMH1 and TMH2 in the WzzE^GG333/4LL^ mutant (Figures 3D and S10A). Alignments of WzzE monomers predicted to closely interact with WzyE show that WzyE rotates in the plane perpendicular to the membrane in the WzyE‐WzzE^GG333/4LL^ model compared to the wild type (Figure S10B,C). The alignment also shows the distance in the structure between the TMH of opposite WzzE monomers was 2.0 to 2.7 Å smaller in the WzzE^GG333/4LL^ model than in the wild‐type model. In compensation, WzyE takes on a more condensed structure (1.4 Å narrower) in the WzzE^GG333/4LL^ model. As this type of rotation and structure change could impair WzyE function, this prediction provides a possible hypothesis that WzzE^GG3334/LL^ has impaired interaction with WzyE. Alternatively, disruption of TMH packing may interfere with an intrinsic and lesser defined activity of WzzE.

In contrast to mutations in TMH2, many substitutions introduced into the periplasmic domain rendered WzzE unstable, as evidenced by loss of chloramphenicol resistance conferred by the WzzE‐Cat fusion. This observation underscores the importance of the periplasmic domain for WzzE's structural integrity and/or multimerization. Prior work on other PCP1s suggests that multiple surface‐exposed residues within the periplasmic domain affect carbohydrate chain‐length regulation, producing either shorter or longer Oag chains (Kalynych, Yao, et al. 2012; Papadopoulos and Morona 2010; Daniels and Morona 1999; Hong and Payne 1997). More recently, increasing evidence suggests that the inner surface of the WzzE octamer may bind to the elongating carbohydrate to facilitate growth of the chain (Wiseman et al. 2023; Hong et al. 2023; Weckener et al. 2023; Tran and Morona 2013).

WzzE's internal cavity possesses two highly concentrated, negatively charged bands, separated by a flexible neutrally charged loop that may bind the growing carbohydrate chain (Wiseman et al. 2023). A loop at the apex of the periplasmic domain has been suggested to “ratchet” up and down to facilitate carbohydrate movement over this binding surface (Wiseman et al. 2023). F104 is buried near the base of the periplasmic “bell” and may affect this binding surface indirectly. In contrast, K162 is positioned adjacent to the lower negatively charged band and L259 residues within the proposed ratchet loop. Mutations to L259 did not affect chain‐length regulation; however, loss of charge at K162 did prevent chain‐length regulation and ECA_CYC_ synthesis, perhaps supporting this model of ECA chain‐length elongation. As to how the *wzzE*_*K162A*_ and *wzzE*_*ΔF104*_ mutations lead to loss of ECA_CYC_ production, we speculate that the chain‐length regulating activity of WzzE—related to the carbohydrate binding and ratcheting mechanism—is necessary for ECA_CYC_ synthesis.

**FIGURE 8:**
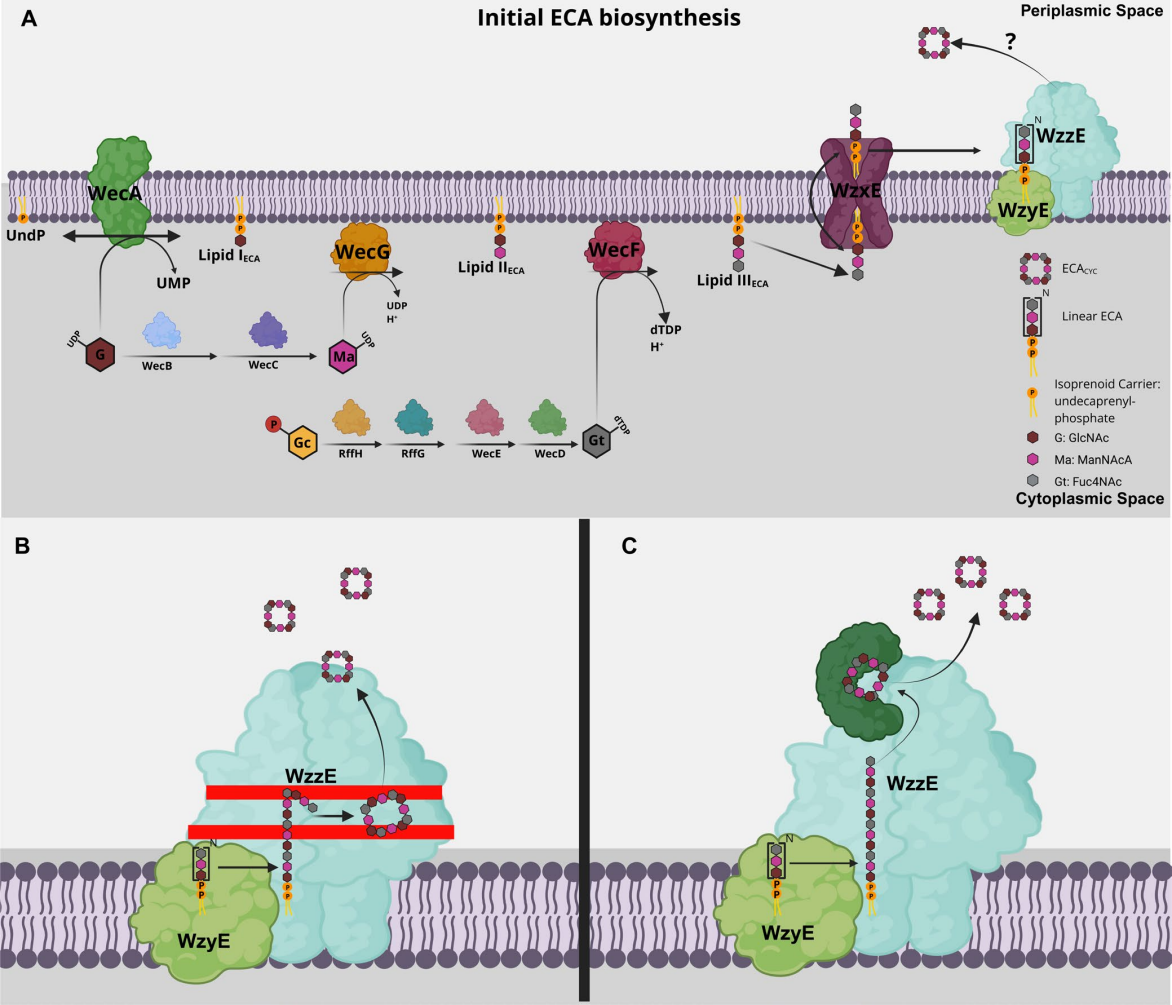
Model for WzzE's function in linear ECA chain‐length regulation and ECA_CYC_ production. (A) The synthesis of ECA_CYC_ begins with WecA which initiates the biosynthesis of ECA and O antigen by catalyzing the transfer of GlcNAc‐1‐phosphate onto an undecaprenyl phosphate carrier. Additional amino sugars are assembled onto the carrier until all ECA sugar units are assembled. The intermediate is then flipped across the IM by WzxE, and WzyE in a complex with WzzE polymerizes the repeating trisaccharide units. WzzE regulates the number of RUs and cyclization of ECA. We hypothesize that WzzE independently regulates ECA_CYC_ biosynthesis through a function distinct from its role in chain length regulation. (B) One possible mechanism for ECA_CYC_ synthesis is WzzE bends ECA molecules of the correct size into an orientation that can be spontaneously cyclized. Red bands on the WzzE structural model highlight two regions of highly concentrated negative charge within the internal cavity, which may promote spontaneous cyclization of the ECA reducing ends. (C) Alternatively, WzzE facilitates the binding of another protein (i.e., a cyclase) that can cyclize ECA chains of the appropriate length. Created in BioRender. Carr (2026) https://BioRender.com/nishj4v.

In contrast, the *wzzE*_*F104H*_ mutation reduces ECA_CYC_ levels while maintaining comparatively wild‐type chain‐length regulation. Moreover, chain‐length regulation by *wzzE*_*F104Y*_ was indistinguishable from *wzzE*_*F104H*_, despite maintaining wildtype levels of ECA_CYC_ production. These results demonstrate that chain‐length regulation alone is insufficient for normal ECA_CYC_ synthesis and suggest that WzzE plays an additional role specific to ECA_CYC_ synthesis beyond that of chain‐length regulation (Figure 8A). Therefore, we propose two models for the role of WzzE in ECA_CYC_ biosynthesis. In the first, WzzE functions as a structural scaffold that imposes conformational constraints on elongating ECA chains, selectively stabilizing polymers of the appropriate length and bending them into a conformation that promotes spontaneous intramolecular cyclization (Figure 8B). We propose that, in this scenario, WzzE‐mediated curvature or spatial positioning of the glycan can bring the reducing and non‐reducing ends into proximity, thereby facilitating ring closure without the requirement of enzymatic activity. Our alternative model is that WzzE acts as a platform for recruitment or stabilization of an accessory factor, such as a cyclase, that specifically recognizes ECA chains of the correct length and catalyzes their conversion into ECA_CYC_ (Figure 8C). In this model, WzzE may regulate substrate specificity by restricting access to chains that meet defined structural or length criteria. We believe that these models are not mutually exclusive and collectively highlight potential mechanisms by which WzzE could coordinate ECA chain length control and ECA_CYC_ production.

Unexpectedly, we consistently observed that WzzE^ΔF104^ and WzzE^GG333/4LL^ mutants had greater linear ECA levels than what was observed in our *wzzE* deletion. We confirmed that this phenotype does not arise from compensation by other PCP1s in *E. coli*, although we did notice a modest increase in linear ECA in strains lacking all other PCP1 genes. Therefore, why WzzE^ΔF104^ and WzzE^GG333/4LL^ have elevated linear ECA levels remains unclear. One possible explanation is that inactive WzzE octamers partially sequester growing ECA chains, increasing variability in chain length. Regardless, we believe that the phenotypic distinction between Δ*wzzE* strain and the mutants strengthens the conclusion that these mutants produce inactive proteins which accumulate but are incapable of regulating ECA chain‐length or supporting ECA_CYC_ biosynthesis, despite the fact that we were not able to directly assess the impact of chromosomally expressed *wzzE*_*ΔF104*_ and *wzzE*_*GG333/4LL*_ on protein accumulation or membrane localization.

Overall, our analysis interrogates only a small fraction of WzzE residues, and additional mutations that selectively impair ECA_CYC_ biosynthesis likely exist. Identification of such mutants will clarify the molecular mechanism of ECA_CYC_ biosynthesis. Nonetheless, our findings reveal fundamental requirements for ECA_CYC_ and chain‐length regulation. With respect to potential broader physiological effects, including changes in membrane permeability or sensitivity to environmental stressors, these analyses could provide additional insight into the consequences of the WzzE variants. We propose coordinated activity between WzyE and WzzE, together with periplasmic charge elements within WzzE that facilitate chain elongation, is required for ECA_CYC_ biogenesis. Importantly, we demonstrate that chain‐length regulation can be genetically uncoupled from ECA_CYC_ synthesis, indicating that WzzE possesses a second function beyond chain‐length regulation that is important for ECA_CYC_ biosynthesis. These insights help to define key features of WzzE that enable synthesis of a cyclic carbohydrate as well as establish a framework for deeper structural analysis of WzzE's role in ECA_CYC_ synthesis and function.

## 4 Experimental Procedures

### 4.1 Strains and Growth Conditions

The strains used in this study are listed in Table S2. Cultures were inoculated and grown at 37°C in LB Lennox (Fisher Bioreagents) unless otherwise noted, and, when necessary, were supplemented with 20 mg/L chloramphenicol, 25 mg L^−1^ kanamycin, 10 mg L^−1^ tetracycline, or a combination thereof (Gold Biotechnology). Insertion–deletion alleles containing kanamycin resistance cassettes from the Keio collection (Baba et al. 2006) were moved to isogenic backgrounds using P1*vir* transduction (Silhavy et al. 1984). Clean deletions were constructed using the Flp recombinase‐FRT system as described (Datsenko and Wanner 2000).

### 4.2 CRISPR‐Cas9 Chromosomal Mutagenesis

Chromosomal mutagenesis in *wzzE* was performed using a CRISPR‐Cas9 system harboring a pCRISPR::protospacer plasmid in W3110 containing a heat shock‐inducible λcI857 lysogen, as previously described (Pyne et al. 2015; Jiang et al. 2013). The pCas9 (Addgene #42876) and pCRISPR (Addgene #42975) plasmids were a kind gift from Professor Luciano Marraffini (Rockefeller University). Oligonucleotides used to generate the protospacer and repair templates used for mutagenesis are listed in Table S3. Prior to chromosomal mutagenesis, *metE*::Tn*10* was linked to *wzzE* (Nichols et al. 1998). Strains carrying *metE*::Tn*10* were supplemented with 1 μm cobalamin (vitamin B_12_) when grown in minimal media (Banerjee et al. 1989). Donor DNA for genome editing was generated by either PCR amplification from pZS21 containing mutated *wzzE or from double stranded Hifi gene fragments (IDTDNA)*. For *wzzE*, a NGG PAM near the native loci was selected to generate a targeted deletion, and the corresponding 30‐nt protospacer was cloned into pCRISPR as has been described (Pyne et al. 2015; Jiang et al. 2013). Correct guide insertion was confirmed by colony PCR and Sanger sequencing using primers flanking ~100 bp up and downstream of *wzzE*. For mutagenesis, the λcI857 lysogen was induced by growth at 42°C, and cells were then transformed with the pCas9 and pCRISPR plasmids as well as the repair template. After mutations were confirmed by PCR and sequencing, the mutated *wzzE* was transferred to a clean MG1655 background via P1*vir* transduction and selecting for the linked Tn*10*. Transfer of the mutation was confirmed by PCR and sequencing.

### 4.3 Plasmids, Cloning, and Site Directed Mutagenesis

All primers used in this study are listed in Table S4. To subclone the p*wzzE* expression plasmid, chromosomal *wzzE* with an ochre stop codon was PCR‐amplified using primers pZS21‐wzzE_fwd and wzzE‐pZS21_rev. The low copy number plasmid pZS21 (Lutz and Bujard 1997) was amplified using primers pZS21_fwd and pZS21_rev. The plasmid was assembled using HiFi Assembly Master Mix (New England Biolabs) according to the manufacturer's instructions. To construct p*wzzE‐cat*, *wzzE* lacking a stop codon was amplified using primers pZS21‐wzzE_fwd and wzzE‐cat_rev. The chloramphenicol acetyltransferase gene (*cat*) was amplified from pBAD33 (Guzman et al. 1995) using primers wzzE‐cat_fwd and cat‐pZS21_rev. These fragments were assembled into pZS21, amplified as described above, using HiFi Assembly. To construct p*wzzE*‐FLAG, p*wzzE* was amplified using primers p*wzzE*_x3F_Fwd and p*wzzE*_x3F_Rvr, which contained sequence homology to a glycine‐serine‐linked FLAG tag that incorporated a stop codon. The glycine‐serine‐linked FLAG tag was supplied as a gene‐block fragment (Table S3) and assembled into the p*wzzE* plasmid via HiFi Assembly. To generate the p*wzzE‐cat‐*FLAG plasmid, *cat* was amplified using primers WzzE:Cmr Fp and WzzE:Cmr Rp and the p*wzzE*‐FLAG plasmid backbone was amplified using Cmr: GS‐x3F_FP and wzzE‐cat_rev. The fragments were assembled using HiFi Assembly.

Site‐directed mutations were generated using Q5 PCR mutagenesis (New England Biolabs). Briefly, non‐overlapping primers containing the mutation of interest were used to amplify the indicated pZS21‐sanA plasmids (pZS21‐*wzzE*, pZS21‐*wzzE‐cat*, and pZS21‐*wzzE*‐FLAG). PCR products were digested with DpnI (New England Biolabs), phosphorylated using T4 polynucleotide kinase (New England Biolabs), and self‐ligated with T4 DNA ligase (New England Biolabs). Ligations were transformed into Turbo cloning cells (New England Biolabs), and the mutations were confirmed by sequencing (Plasmidsaurs). Primers used for all site‐directed mutants are listed in Table S4. Modeling of the predicted mutant structures was performed using AlphaFold 3 (Abramson et al. 2024) with eight monomers of WzzE and one of WzyE. Relevant confidence scores are given in Table S1 and Figure S5.

### 4.4 Antibiotic Sensitivity

Efficiency of plating (EOP) assays were performed by plating 10‐fold serial dilutions of overnight cultures onto LB plates containing the indicated antibiotics using a 48‐pin tool. Plates were incubated overnight at 30°C before imaging.

### 4.5 Immunoblot Analysis

Immunoblots for WzzE‐FLAG and ECA were conducted as previously described with minor modification (Mitchell et al. 2018; Morris and Mitchell 2023). For analysis of WzzE‐FLAG protein levels, samples were normalized to equal OD_600_ from overnight cultures, lysed by boiling for 5 min in sample loading buffer, and loaded on 12% SDS‐PAGE gels. Levels of WzzE‐FLAG were quantitated by densitometry using ImageJ (Crawford et al. 2012) as has been previously described (Mitchell et al. 2018) on immunoblots with the lowest exposure at which WzzE bands could be detected. Samples for ECA analysis were prepared and immunoblotted as previously described with minor modification (Mitchell et al. 2018; Morris and Mitchell 2023). To avoid ECA degradation, ultrapure water, commercial lysis reagents (BugBuster, Millipore Sigma), commercial TRIS‐TRICINE‐SDS running buffer (National Diagnostics Supplier), and precast gels were used for ECA immunoblots. In addition, all western equipment was washed before and after ECA immunoblots with Extran 300 Detergent (Millipore Sigma) and rinsed 10 times with ultrapure water. To determine whether proteins affect the apparent chain length of ECA, we pre‐treated samples with ProK (Qiagen). Samples were subjected to different pre‐treatment conditions, varying both in the concentration of ProK and time of treatment (Figure S8A), based on protocols used in previous (Peters et al. 1985; Liu et al. 2020; Leo, Tran, and Morona 2021; Maczuga, Tran, and Morona 2022) papers. All treatments were performed at 56°C. Following ProK treatment, samples were boiled again for 10 min and loaded on 15% SDS‐PAGE gels. The outcomes of each treatment condition and their respective effects on the resolution and clarity of the immunoblot, as well as effects on ECA levels are shown in (Figure S8B). Antibodies used for blotting were as follows: M2 αFLAG (Millipore Sigma, 1:50,000 dilution), αGroEL (Millipore Sigma, 1:60,000 dilution), αECA (a gift from Professor Renato Morona, University of Adelaide, 1:30,000), rabbit α‐mouse (Prometheus, 1:100,000 dilution), goat α‐rabbit (Prometheus, 1:100,000 dilution), or αBamA (a gift from Professor Thomas Silhavy, Princeton University, 1:50,000 dilution).

### 4.6 Quantification of Cyclic ECA

Quantification of ECA_CYC_ was performed as previously described, with minor modifications (Mitchell et al. 2018). Briefly, cells were grown in M63 glucose minimum media lacking nitrogen and supplemented with either 0.2% (^14^NH_4_)_2_SO_4_ or (^15^NH_4_)_2_SO_4_ (Cambridge Isotope Laboratories). ECA_CYC_ was purified as previously described. Sample analysis was performed using a Thermo Scientific Q Exactive Focus and liquid chromatography unit (UltiMate 3000 RS). Purified samples were dissolved in 0.1% formic acid and separated by injecting 20 μL of sample into the Synergi Hydro‐RP (4.6 × 250 mm; 4.0 μm) C18 column (*Phenomenex*). The mobile phase consisted of 0.1% formic acid (eluent A) and acetonitrile (eluent B). The flow rate was set at 800 μL/min with the following gradient: 0–2.0 min 5% B, 2.0–12.0 min 5%–90% B, 12–14 min 90% B, and returned to 5% B in 0.1 min and held for *4* min at 5% B. The Q Exactive Focus ESI source was operated in full MS (1000–3000 m/z). The mass resolution was tuned to 70,000 FWHM at m/z 200. The spray voltage was set to 3.5 kV in negative mode, and the sheath gas and auxiliary gas flow rates were set to 60 and 20 arbitrary units, respectively. The transfer of capillary and auxiliary gas heater temperatures was held at 380°C and 300°C, respectively. The S‐Lens RF level was set at 70 V and maximum injection time was set to 250 ms. Tandem mass analysis was performed in the HCD cell at 60 V for the m/z 1213.4 and 1219.4 for the labeled and unlabeled ions, respectively. The MS2 resolution was set to 35,000 with the quadrupole isolation window of 0.4 m/z. For relative quantification, the integrated peak‐area ratios of the extracted‐ion chromatograms (for m/z 1213.4 and 1219.4) were used, and the ratio of the two areas was calculated. The Exactive Series 2.11/Xcalibur 4.2.47 software was used for data acquisition and processing.

## Author Contributions

**Jennifer S. Rudolf:** investigation, writing – review and editing, visualization. **Daniel J. Warzecha:** investigation, visualization, writing – review and editing. **Joseph F. Carr:** conceptualization, investigation, writing – original draft, visualization, writing – review and editing. **Yohannes H. Rezenom:** conceptualization, investigation, visualization, writing – review and editing, writing – original draft, methodology. **Angela M. Mitchell:** conceptualization, funding acquisition, writing – review and editing, visualization, project administration, supervision.

## Funding

This work was supported by National Institute of Allergy and Infectious Diseases (R01‐AI155915).

## Ethics Statement

The work was performed under the approval of the Texas A&M University institutional Biosafety Committee.

## Conflicts of Interest

The authors declare no conflicts of interest.

## Supporting information


**Figure S1:** Structure of WzzE. (A) A cryoEM structure of the WzzE octamer (1) is shown with alternating monomers indicated by color. (B) The locations of residues changed in this study are called out in magenta on a WzzE monomer.
**Figure S2:** Liquid chromatography‐mass spectrometry (LC–MS) quantification of ECACYC. (A) ECA immunoblot analysis for the indicated strains. Deletion of wecH had no observable effect on linear ECA distribution relative to wild type, whereas loss of wzzE resulted in clear dysregulation of chain length, indicating that wecH does not affect linear ECA regulation. However, deleting wecH does somewhat reduce linear ECA levels in both the wild‐type and wzzE mutant strains. (B) A crystal structure image of ECACYC in its square conformation (2) is shown with its chemical formula. The 12 nitrogen atoms (highlighted) are made 1 Da heavier by growth with ^15^N, shifting the mass of ECACYC by 12 Da. (C) A MS trace for a mixture of ECACYC from cells grown in normal nitrogen (^14^N) and in heavy nitrogen (^15^N) along with simulated traces of the molecular species for each of these molecules. The experimental data match the simulated results. (D) Representative MS traces are shown for samples from the indicated strains mixed before the start of ECACYC purification. The ratio of the ^14^N and ^15^N peaks are similar whether the sample grown in ^14^N carries the pwzzE (Figure 1D) or pwzzE 2 cat plasmid. (E) Representative LC traces are shown for purified ECACYC samples from the indicated strains. Data shown in black represent ΔwzzE ΔwecH strains carrying pZS21, while red is complemented with pwzzE and labeled. The bottom panel overlays the two traces showing loss of the ECACYC peak in the uncomplemented strain.
**Figure S3:** ChemDraw Representation of ECACYC Fragmentation. (A) Structural representation of ECACYC generated in ChemDraw depicting individual sugar fragment structures corresponding to observed fragmentation events. Fragmentation sites are color coded and have been assigned letter identifiers that correspond directly to the LC–MS/MS spectra and fragmentation assignments shown in the main text.
**Figure S4:** Levels, stability, and multimerization of wzzE mutants. (A) The effect of the indicated mutations to the region of wzzE encoding TMH2 made in the pwzzE‐cat‐FLAG background was assayed by immunoblotting. BamA serves as a loading control. All mutants appear to be equally stable. (B) EOPs are shown assaying the stability and multimerization of WzzE using the proxy of chloramphenicol resistance. Mutations are expressed from the pwzzE‐cat construct. All mutants retain equal chloramphenicol resistance. (C) The effect of the indicated mutations to the region of wzzE encoding the periplasmic domain made in the pwzzE‐cat‐FLAG background was assayed by immunoblotting. BamA serves as a loading control. All mutants except wzzEΔF104 appear to be equally stable. The C‐terminal tags are largely cleaved from WzzEΔF104 making its stability unclear. (D) EOPs are shown assaying the stability and multimerization of WzzE using the proxy of chloramphenicol resistance in mutants expressed from the pwzzE‐cat construct. Several mutants show decreased chloramphenicol resistance, including F104Y, K162A, and L259A.
**Figure S5:** Confidence scores for WzzE‐WzyE complex structural prediction. Predicted local distance difference test (pLDDT) scores are shown for WzyE and the first WzzE monomer from an AlphaFold 3 (3) prediction of the complex of wild‐type WzzE(8) with WzyE. Distributions were similar for the predicted structures with WzzE mutants.
**Figure S6:** Changes to the periplasmic domain of WzzE are prone to induce instability and/or affect multimerization. Strains with the indicated plasmids were assayed for chloramphenicol resistance as a measure of their stability and ability to multimerize. These periplasmic mutations led to loss of chloramphenicol resistance indicating that the mutant proteins were unstable or unable to multimerize.
**Figure S7:** The effect of WecH on linear ECA chain length regulation. (A) ECA immunoblot analysis showing the effect of chromosomal wzzE mutations on ECA chain length regulation in a ΔwecH background. (B) CFU/mL and GroEL immunoblotting to show consistent normalization of wzzE chromosomal mutant strains.
**Figure S8:** The effect of proteinase K treatment on ECA immunoblots. (A) A summary of proteinase K treatment conditions prior to ECA immunoblotting is shown. Five treatments (T1–T5) conditions were chosen from or modified from literature and varied in proteinase K concentration added and time of digestion at 56°C. (B) Immunoblot analysis of ECA following treatment. Whole‐cell lysates from wild‐type and ΔwzzE were subjected to the treatments described above and probed with anti‐ECA antibodies. For each treatment, the wild‐type and ΔwzzE samples were processed and run in parallel to compare the impact of proteinase K treatment on the banding pattern and ECA stability. The far‐left lane (“w/o T”) represents an untreated wild‐type control, harvested via our normal protocol. Longer treatments resulted in a lower signal ECA signal, likely because of ECA degradation. However, a short incubation with high amounts of proteinase K resulted in more even banding without significant loss of ECA.
**Figure S9:** Relative quantification of linear ECA chain length. ECA immunoblot signals were quantified using ImageJ by partitioning bands into low, medium, and high chain lengths. The relative abundance of each subunit class was quantified by normalizing signal intensity within each sample and expressing each fraction as a percentage of the total ECA in that sample. Statical significance between each mutant's subunit class was compared to wild type. Data are the average of three biological replicates ± SEM. *p < 0.05 by Mann–Whitney test.
**Figure S10:** WzzE‐WzyE interactions may shift in the WzzEGG333/4LL mutant. Models of the WzzE(8)‐WzyE complex were constructed with Alphafold3 for wild‐type WzzE and the WzzEGG333/4LL mutant and the WzzE monomers most closely contacting WzyE were aligned. (A) The TMHs of WzzE are closer together in the wild‐type model than in the WzzEGG333/4LL model. (B‐C) The aligned WzzE monomers are shown with WzyE (B) or the aligned monomers and the opposite monomers (C). The predicted angle of WzyE shifts in the WzzEGG333/4LL model compared with the wild type.
**Table S1:** Confidence scores for WzzE‐WzyE structural predictions.
**Table S2:** Strains used in this study.
**Table S3:** CRISPR spacer oligos and repair templates.
**Table S4:** Primers used in this study.
**Dataset: S1** Ion fragmentation data for LC–MS/MS of ECACYC.

## Data Availability

The data that supports the findings of this study are available in manuscript and in the Supporting Information.
