# Supplementary material for: Chain‐Length Regulation by WzzE Is Necessary for, but Genetically Separable From, Cyclic Enterobacterial Common Antigen Synthesis
Source: Mol Microbiol. 2026 Jun 19;126(2):190–207. doi: 10.1111/mmi.70088 (PMC13432762; doi:10.1111/mmi.70088)
Supplement: Supplementary file 1 — Figure S1: Structure of WzzE. (A) A cryoEM structure of the WzzE octamer (1) is shown with alternating monomers indicated by color. (B) The locations of residues changed in this study are called out in magenta on a WzzE monomer. Figure S2: Liquid chromatography‐mass spectrometry (LC–MS) quantification of ECACYC. (A) ECA immunoblot analysis for the indicated strains. Deletion of wecH had no observable effect on linear ECA distribution relative to wild type, whereas loss of wzzE resulted in clear dysregulation of chain length, indicating that wecH does not affect linear ECA regulation. However, deleting wecH does somewhat reduce linear ECA levels in both the wild‐type and wzzE mutant strains. (B) A crystal structure image of ECACYC in its square conformation (2) is shown with its chemical formula. The 12 nitrogen atoms (highlighted) are made 1 Da heavier by growth with 15N, shifting the mass of ECACYC by 12 Da. (C) A MS trace for a mixture of ECACYC from cells grown in normal nitrogen (14N) and in heavy nitrogen (15N) along with simulated traces of the molecular species for each of these molecules. The experimental data match the simulated results. (D) Representative MS traces are shown for samples from the indicated strains mixed before the start of ECACYC purification. The ratio of the 14N and 15N peaks are similar whether the sample grown in 14N carries the pwzzE (Figure 1D) or pwzzE 2 cat plasmid. (E) Representative LC traces are shown for purified ECACYC samples from the indicated strains. Data shown in black represent ΔwzzE ΔwecH strains carrying pZS21, while red is complemented with pwzzE and labeled. The bottom panel overlays the two traces showing loss of the ECACYC peak in the uncomplemented strain. Figure S3: ChemDraw Representation of ECACYC Fragmentation. (A) Structural representation of ECACYC generated in ChemDraw depicting individual sugar fragment structures corresponding to observed fragmentation events. Fragmentation sites are color coded and have b [file MMI-126-190-s001.pdf]

SUPPLEMENTARY INFORMATION FOR

Chain-length regulation by WzzE is necessary for, but genetically separable from, cyclic enterobacterial common antigen synthesis

Joseph F. Carr[a], Yohannes H. Rezenom[b]*, Jennifer S. Rudolf[a]*, Daniel J. Warzecha[a]*, & Angela M. Mitchell[a]#

[a] Department of Biology, Texas A&M University, College Station, TX
[b] Department of Chemistry, Texas A&M University, College Station, TX

* YHR, JSR, and DJW contributed equally to this work.

#Address correspondence to Angela M. Mitchell, amitchell@bio.tamu.edu

SUPPLEMENTAL FIGURES

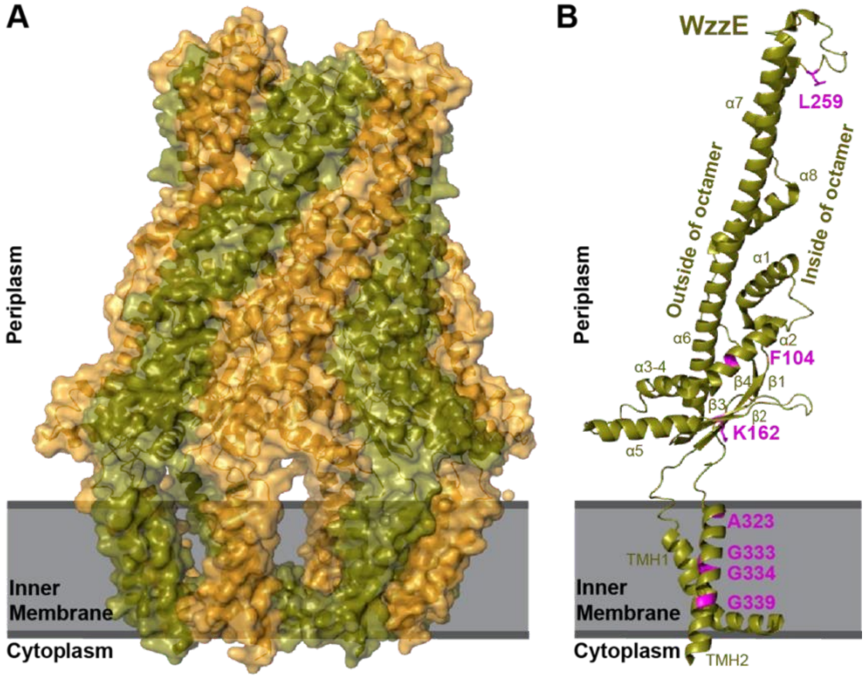

Figure S1. Structure of WzzE. (A) A cryoEM structure of the WzzE octamer (1) is shown with alternating monomers indicated by color. **(B)** The locations of residues changed in this study are called out in magenta on a WzzE monomer.

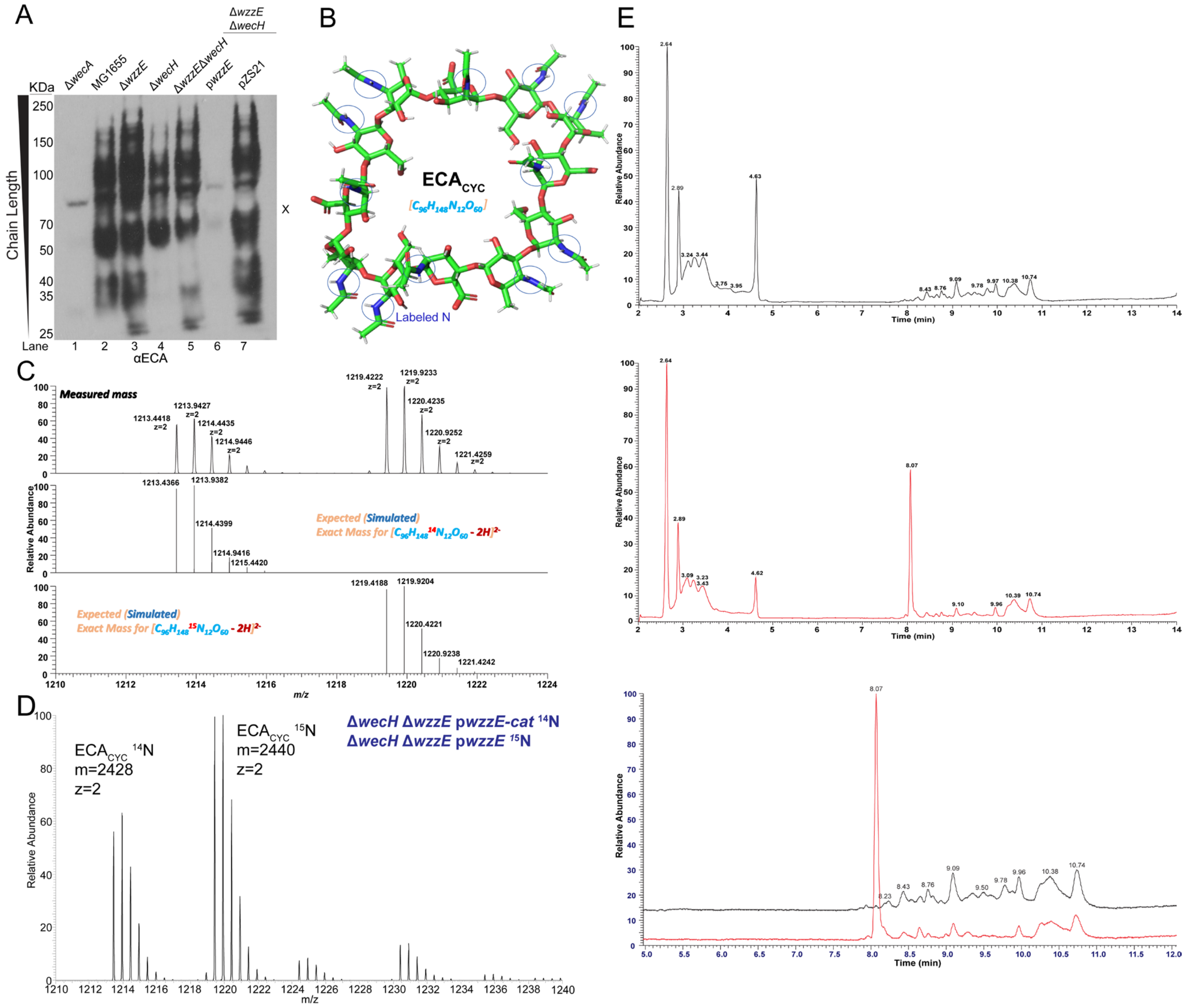

Figure S2. Liquid chromatography-mass spectrometry (LC-MS) quantification of ECA$_{CYC}$. (A) ECA immunoblot analysis for the indicated strains. Deletion of *wecH* had no observable effect on linear ECA distribution relative to wild type, whereas loss of *wzzE* resulted in clear dysregulation of chain length, indicating that *wecH* does not affect linear ECA regulation. However, deleting *wecH* does somewhat reduce linear ECA levels in both the wild-type and *wzzE* mutant strains. **(B)** A crystal structure image of ECA$_{CYC}$ in its square conformation (2) is shown with its chemical formula. The 12 nitrogen atoms (highlighted) are made 1 Da heavier by growth with ^{15}N, shifting the mass of ECA$_{CYC}$ by 12 Da. **(C)** A MS trace for a mixture of ECA$_{CYC}$ from cells grown in normal nitrogen (^{14}N) and in heavy nitrogen (^{15}N) along with simulated traces of the molecular species for each of these molecules. The experimental data match the simulated results. **(D)** Representative MS traces are shown for samples from the indicated strains mixed before the start of ECA$_{CYC}$ purification. The ratio of the ^{14}N and ^{15}N peaks are similar whether the sample grown in ^{14}N carries the p*wzzE* (Figure 1D) or p*wzzE*-

cat plasmid. **(E)** Representative LC traces are shown for purified ECA_{CYC} samples from the indicated strains. Data shown in black represent Δ*wzzE* Δ*wecH* strains carrying pZS21, while red is complemented with p*wzzE* and labeled. The bottom panel overlays the two traces showing loss of the ECA_{CYC} peak in the uncomplemented strain.

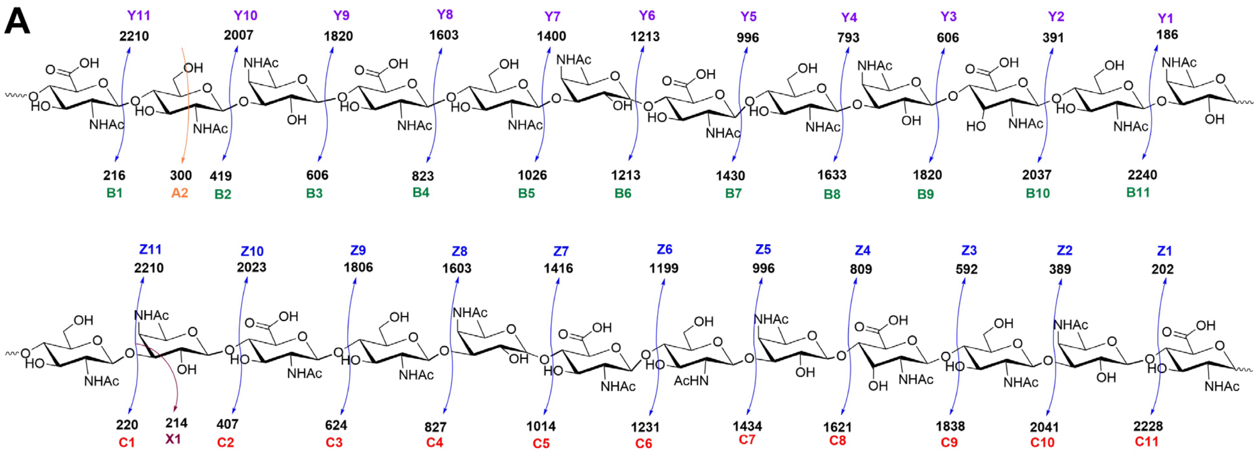

Figure S3. ChemDraw Representation of ECA_{CYC} Fragmentation. (A) Structural representation of ECA_{CYC} generated in ChemDraw depicting individual sugar fragment structures corresponding to observed fragmentation events. Fragmentation sites are color-coded and have been assigned letter identifiers that correspond directly to the LC-MS/MS spectra and fragmentation assignments shown in the main text.

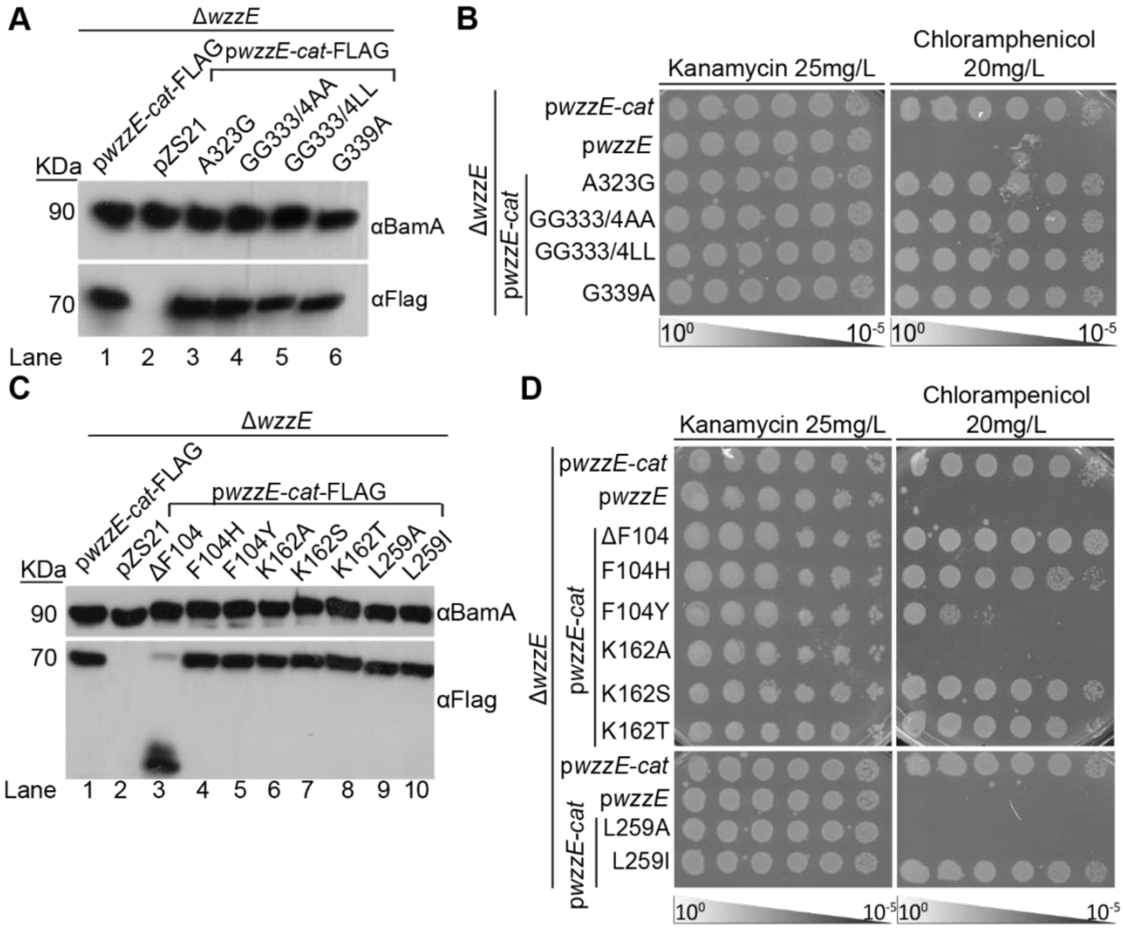

Figure S4. Levels, stability, and multimerization of *wzzE* mutants. (A) The effect of the indicated mutations to the region of *wzzE* encoding TMH2 made in the p*wzzE-cat*-FLAG background was assayed by immunoblotting. BamA serves as a loading control. All mutants appear to be equally stable. **(B)** EOPs are shown assaying the stability and multimerization of WzzE using the proxy of chloramphenicol resistance. Mutations are expressed from the p*wzzE-cat* construct. All mutants retain equal chloramphenicol resistance. **(C)** The effect of the indicated mutations to the region of *wzzE* encoding the periplasmic domain made in the p*wzzE-cat*-FLAG background was assayed by immunoblotting. BamA serves as a loading control. All mutants except $wzzE_{\Delta F104}$ appear to be equally stable. The C-terminal tags are largely cleaved from $\text{WzzE}^{\Delta F104}$ making its stability unclear. **(D)** EOPs are shown assaying the stability and multimerization of WzzE using the proxy of chloramphenicol resistance in mutants expressed from the p*wzzE-cat* construct. Several mutants show decreased chloramphenicol resistance, including F104Y, K162A, and L259A.

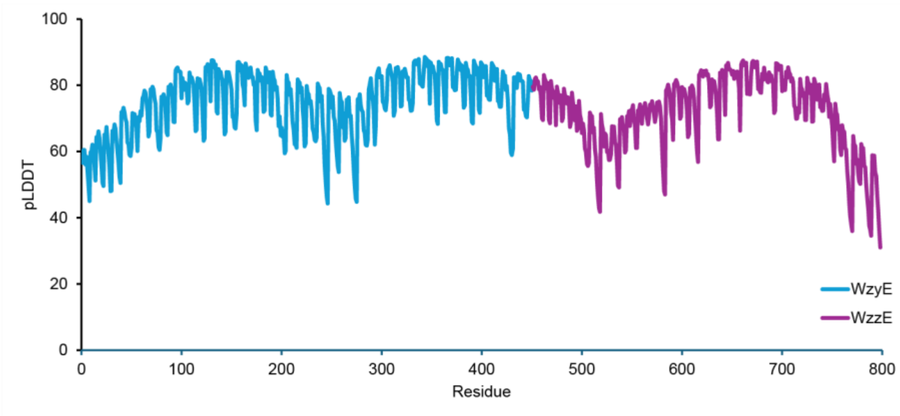

Figure S5. Confidence scores for WzzE-WzyE complex structural prediction. Predicted local distance difference test (pLDDT) scores are shown for WzyE and the first WzzE monomer from an AlphaFold 3 (3) prediction of the complex of wild-type WzzE(8) with WzyE. Distributions were similar for the predicted structures with WzzE mutants.

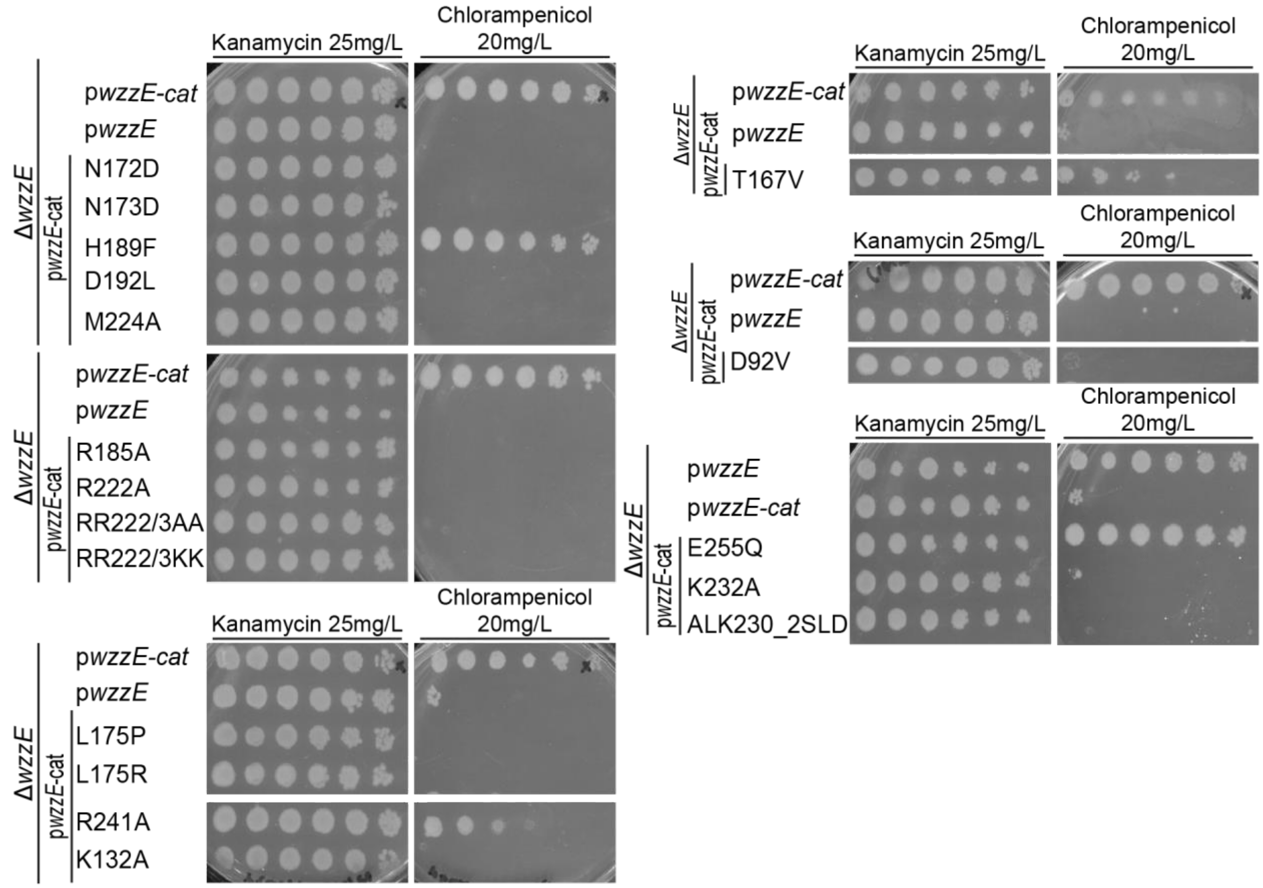

Figure S6. Changes to the periplasmic domain of WzzE are prone to induce instability and/or affect multimerization. Strains with the indicated plasmids were assayed for chloramphenicol resistance as a measure of their stability and ability to multimerize. These periplasmic mutations led to loss of chloramphenicol resistance indicating that the mutant proteins were unstable or unable to multimerize.

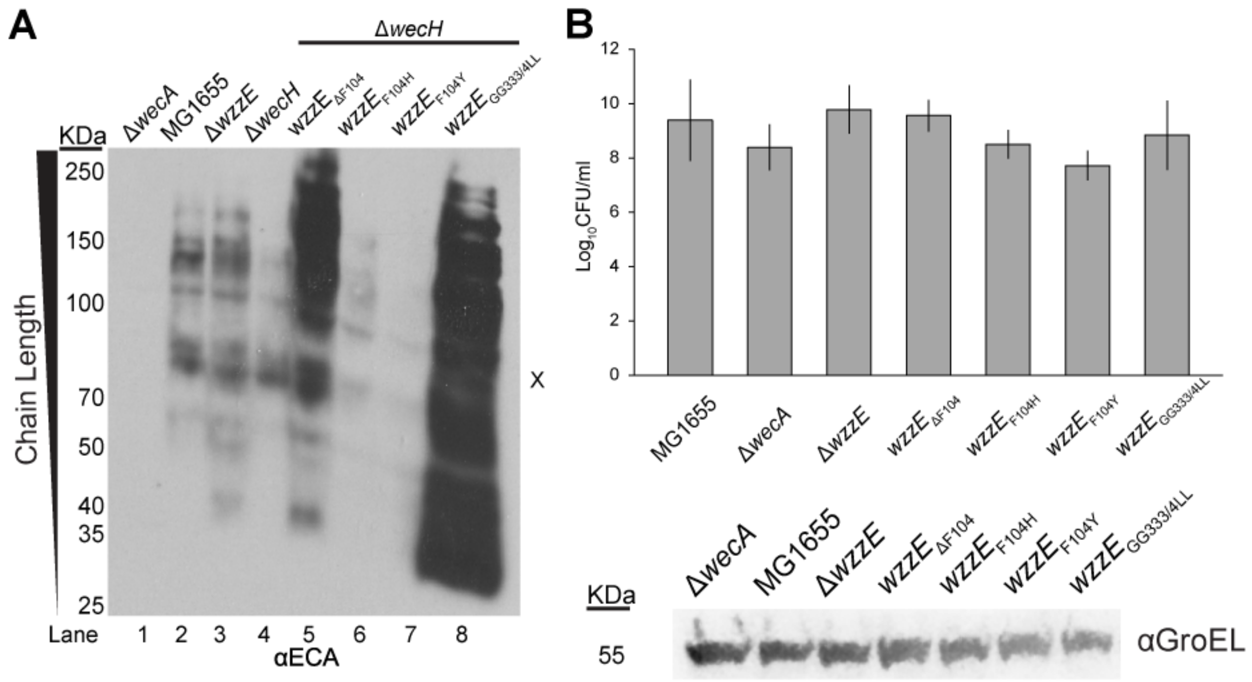

Figure S7. The effect of WecH on linear ECA chain length regulation. (A) ECA immunoblot analysis showing the effect of chromosomal *wzzE* mutations on ECA chain length regulation in a Δ*wecH* background. **(B)** CFU/mL and GroEL immunoblotting to show consistent normalization of *wzzE* chromosomal mutant strains.

A

Treatment	Protinase K concentration	Incubation time
T1	1.6 mg/ml	overnight
T2	0.02 mg/ml	overnight
T3	0.25 mg/ml	overnight
T4	2.5 mg/ml	2 hours
T5	2.5 mg/ml	1 hour

B

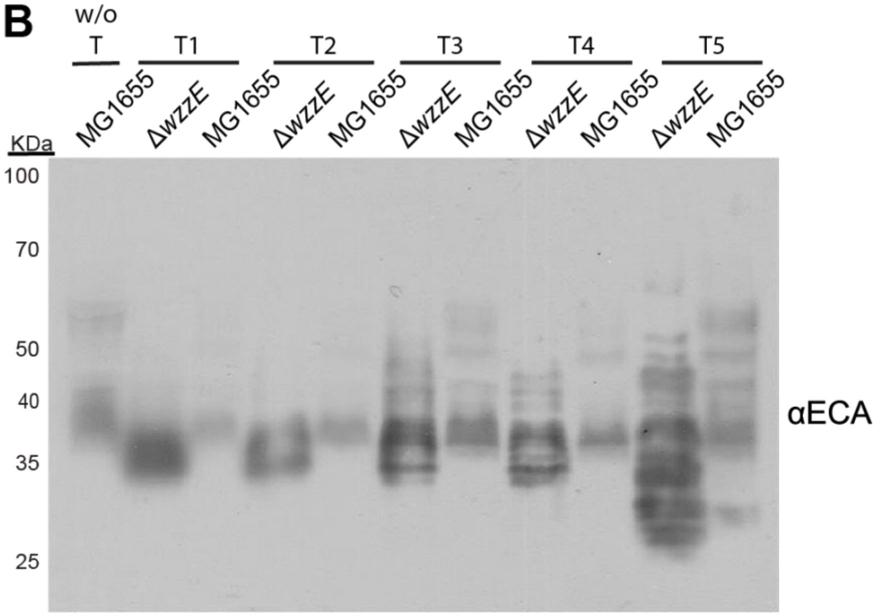

Figure S8. The effect of proteinase K treatment on ECA immunoblots. (A) A summary of proteinase K treatment conditions prior to ECA immunoblotting is shown. Five treatments (T1–T5) conditions were chosen from or modified from literature and varied in proteinase K concentration added and time of digestion at 56 °C. **(B)** Immunoblot analysis of ECA following treatment. Whole-cell lysates from wild-type *and* Δ*wzzE* were subjected to the treatments described above and probed with anti-ECA antibodies. For each treatment, the wild-type and ΔwzzE samples were processed and run in parallel to compare the impact of proteinase K treatment on the banding pattern and ECA stability. The far-left lane ("w/o T") represents an untreated wild-type control, harvested via our normal protocol. Longer treatments resulted in a lower signal ECA signal, likely because of ECA degradation. However, a short incubation with high amounts of proteinase K resulted in more even banding without significant loss of ECA.

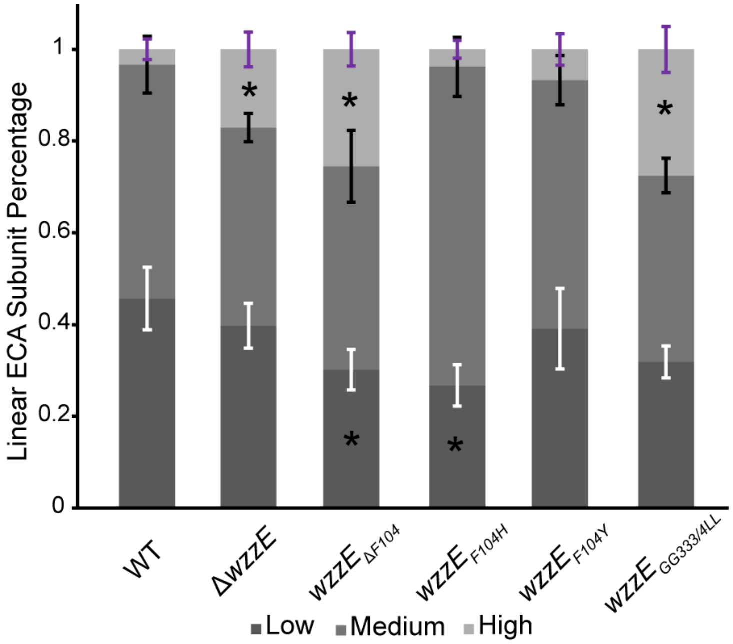

Figure S9. Relative quantification of linear ECA chain length. ECA immunoblot signals were quantified using ImageJ by partitioning bands into low, medium, and high chain lengths. The relative abundance of each subunit class was quantified by normalizing signal intensity within each sample and expressing each fraction as a percentage of the total ECA in that sample. Statical significance between each mutant's subunit class was compared to wild type. Data are the average of three biological replicates ± SEM. * $p<0.05$ by Mann-Whitney test.

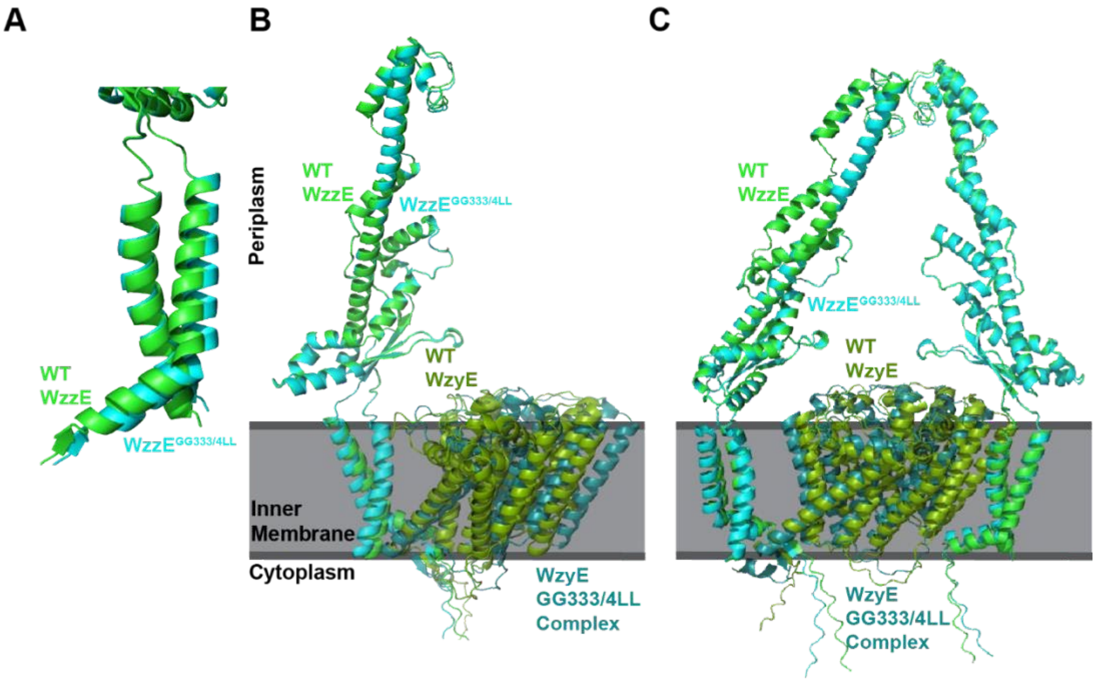

Figure S10. WzzE-WzyE interactions may shift in the WzzE$^{GG333/4LL}$ mutant. Models of the WzzE(8)-WzyE complex were constructed with Alphafold3 for wild-type WzzE and the WzzE$^{GG333/4LL}$ mutant and the WzzE monomers most closely contacting WzyE were aligned. **(A)** The TMHs of WzzE are closer together in the wild-type model than in the WzzE$^{GG333/4LL}$ model. **(B-C)** The aligned WzzE monomers are shown with WzyE (B) or the aligned monomers and the opposite monomers (C). The predicted angle of WzyE shifts in the WzzE$^{GG333/4LL}$ model compared with the wild type.

SUPPLEMENTAL TABLES

Table S1: Confidence scores for WzzE-WzyE structural predictions

**Model**	**pTM[1]**	**ipTM[2]**
**WzzE(8)-WzyE**	0.75	0.74
**WzzE$^{GG333/4LL}$-WzyE**	0.74	0.73
**WzzE$^{F104\Delta}$-WzyE**	0.73	0.71
**WzzE^{F104H}-WzyE**	0.75	0.74
**WzzE^{F104Y}-WzyE**	0.75	0.74
**WzzE(8) only**	0.80	0.78

[1] Predicted modeling template score, ranging from 0-1, indicating the confidence in the overall structure of the complex.

[2] Interface predicted template modeling score, ranging from 0-1, indicating the confidence that relative position of the complex subunits is correct.

Table S2: Strains used in this study

Strain	Genotype	Reference
MG1655	K-12 F^- λ^- *rph-1*	(4)
AM369	MG1655 Δ*wzzE* Δ*yhdP*	(5)
AM365	MG1655 Δ*wzzE*	(5)
AM182	MG1655 Δ*yhdP*	(5)
AM1113	MG1655 Δ*wzzE* Δ*yhdP* pZS21	This study
AM1114	MG1655 Δ*wzzE* Δ*yhdP* *pwzzE*	This study
AM1116	MG1655 Δ*wzzE* Δ*yhdP* p*wzzE-cat*	This study
JC118	MG1655 Δ*wzzE* Δ*yhdP* *pwzzE-FLAG*	This study
JC205	MG1655 Δ*wzzE* pZS21	This study
JC206	MG1655 Δ*wzzE* p*wzzE*	This study
JC207	MG1655 Δ*wzzE* p*wzzE*-FLAG	This study
JC155	MG1655 Δ*wzzE* Δ*wecH*	This study
JC110	MG1655 Δ*wecH*	This study
JC159	MG1655 Δ*wzzE* Δ*wecH* pZS21	This study
JC160	MG1655 Δ*wzzE* ΔwecH p*wzzE*	This study
AM335	MG1655 Δ*wecA*	This study
JC225	MG1655 Δ*wzzE* p$wzzE_{A323G}$	This study
JC226	MG1655 Δ*wzzE* p$wzzE_{GG333/4AA}$	This study
JC227	MG1655 Δ*wzzE* p$wzzE_{GG333/4LL}$	This study
JC228	MG1655 Δ*wzzE* p$wzzE_{G339A}$	This study
JC35	MG1655 Δ*wzzE* p$wzzE_{GG333/4AA}$-*cat*	This study
JC36	MG1655 Δ*wzzE* p$wzzE_{A323G}$-*cat*	This study
JC40	MG1655 Δ*wzzE* p$wzzE_{GG333/4LL}$-*cat*	This study
JC41	MG1655 Δ*wzzE* p$wzzE_{G339A}$-*cat*	This study
JC210	MG1655 Δ*wzzE* p$wzzE_{A323G}$-FLAG	This study
JC211	MG1655 Δ*wzzE* p$wzzE_{GG333/4AA}$-FLAG	This study
JC212	MG1655 Δ*wzzE* p$wzzE_{GG333/4LL}$-FLAG	This study
JC213	MG1655 Δ*wzzE* p$wzzE_{G339A}$-FLAG	This study
JC381	MG1655 Δ*wzzE* p$wzzE_{A323G}$-*cat*-FLAG	This study
JC382	MG1655 Δ*wzzE* p$wzzE_{GG333/4AA}$-*cat*-FLAG	This study
JC383	MG1655 Δ*wzzE* p$wzzE_{GG333/4LL}$-*cat*-FLAG	This study
JC385	MG1655 Δ*wzzE* p$wzzE_{G339A}$-*cat*-FLAG	This study
JC163	MG1655 Δ*wzzE* Δ*wecH* p$wzzE_{GG333/4LL}$	This study
JC167	MG1655 Δ*wzzE* Δ*wecH* p$wzzE_{A323G}$	This study
JC168	MG1655 Δ*wzzE* Δ*wecH* p$wzzE_{GG333/4AA}$	This study
JC170	MG1655 Δ*wzzE* Δ*wecH* p$wzzE_{G339A}$	This study
JC44	MG1655 Δ*wzzE* p$wzzE_{\Delta F104}$-*cat*	This study
JC45	MG1655 Δ*wzzE* p$wzzE_{F104H}$-*cat*	This study

Strain	Genotype	Reference
JC46	MG1655 Δ*wzzE* p*wzzE*$_{F104Y}$-*cat*	This study
JC47	MG1655 Δ*wzzE* p*wzzE*$_{L259A}$-*cat*	This study
JC48	MG1655 Δ*wzzE* p*wzzE*$_{L259I}$-*cat*	This study
JC50	MG1655 Δ*wzzE* p*wzzE*$_{K162A}$-*cat*	This study
JC51	MG1655 Δ*wzzE* p*wzzE*$_{K162S}$-*cat*	This study
JC52	MG1655 Δ*wzzE* p*wzzE*$_{K162T}$-*cat*	This study
JC208	MG1655 Δ*wzzE* p*wzzE*$_{\Delta F104}$-FLAG	This study
JC209	MG1655 Δ*wzzE* p*wzzE*$_{F104H}$-FLAG	This study
JC214	MG1655 Δ*wzzE* p*wzzE*$_{F104Y}$-FLAG	This study
JC215	MG1655 Δ*wzzE* p*wzzE*$_{K162A}$-FLAG	This study
JC216	MG1655 Δ*wzzE* p*wzzE*$_{K162S}$-FLAG	This study
JC217	MG1655 Δ*wzzE* p*wzzE*$_{K162T}$-FLAG	This study
JC218	MG1655 Δ*wzzE* p*wzzE*$_{L259A}$-FLAG	This study
JC219	MG1655 Δ*wzzE* p*wzzE*$_{L259I}$-FLAG	This study
JC226	MG1655 Δ*wzzE* p*wzzE*$_{\Delta F104}$	This study
JC227	MG1655 Δ*wzzE* p*wzzE*$_{F104Y}$	This study
JC228	MG1655 Δ*wzzE* p*wzzE*$_{F104H}$	This study
JC221	MG1655 Δ*wzzE* p*wzzE*$_{K162A}$	This study
JC220	MG1655 Δ*wzzE* p*wzzE*$_{K162S}$	This study
JC231	MG1655 Δ*wzzE* p*wzzE*$_{K162T}$	This study
JC229	MG1655 Δ*wzzE* p*wzzE*$_{L259A}$	This study
JC230	MG1655 Δ*wzzE* p*wzzE*$_{L259I}$	This study
JC376	MG1655 Δ*wzzE* p*wzzE*-*cat*-FLAG	This study
JC373	MG1655 Δ*wzzE* p*wzzE*$_{\Delta F104}$-*cat*-FLAG	This study
JC374	MG1655 Δ*wzzE* p*wzzE*$_{F104H}$-*cat*-FLAG	This study
JC375	MG1655 Δ*wzzE* p*wzzE*$_{F104Y}$-*cat*-FLAG	This study
JC380	MG1655 Δ*wzzE* p*wzzE*$_{L259A}$-*cat*-FLAG	This study
JC384	MG1655 Δ*wzzE* p*wzzE*$_{L259I}$-*cat*-FLAG	This study
JC377	MG1655 Δ*wzzE* p*wzzE*$_{K162A}$-*cat*-FLAG	This study
JC378	MG1655 Δ*wzzE* p*wzzE*$_{K162S}$-*cat*-FLAG	This study
JC379	MG1655 Δ*wzzE* p*wzzE*$_{K162T}$-*cat*-FLAG	This study
JC171	MG1655 Δ*wzzE* Δ*wecH* p*wzzE*$_{\Delta F104}$	This study
JC172	MG1655 Δ*wzzE* Δ*wecH* p*wzzE*$_{F104H}$	This study
JC173	MG1655 Δ*wzzE* Δ*wecH* p*wzzE*$_{F104Y}$	This study
JC174	MG1655 Δ*wzzE* Δ*wecH* p*wzzE*$_{K162A}$	This study
JC175	MG1655 Δ*wzzE* Δ*wecH* p*wzzE*$_{K162S}$	This study
JC176	MG1655 Δ*wzzE* Δ*wecH* p*wzzE*$_{L259A}$	This study
JC177	MG1655 Δ*wzzE* Δ*wecH* p*wzzE*$_{K162T}$	This study
JC178	MG1655 Δ*wzzE* Δ*wecH* p*wzzE*$_{L259I}$	This study

Strain	Genotype	Reference
JC308	MG1655 *wzzE*$_{\Delta F104}$ Δ*wecH*	This study
JC312	MG1655 *wzzE*$_{GG333/4LL}$	This study
JC288	MG1655 *wzzE*$_{GG333/4LL}$ Δ*wecH*	This study
JC321	MG1655 *wzzE*$_{F104Y}$ Δ*wecH*	This study
JC322	MG1655 *wzzE*$_{F104H}$ Δ*wecH*	This study
JC324	MG1655 *wzzE*$_{F104H}$	This study
JC325	MG1655 *wzzE*$_{F104Y}$	This study
JC326	MG1655 *wzzE*$_{\Delta F104}$	This study
JC340	MG1655 Δ*wzzB*	This study
JC341	MG1655 Δ*fepE*	This study
JC342	MG1655 Δ*wzzE* Δ*wzzB:*Kan	This study
JC343	MG1655 Δ*wzzE* Δ*fepE:*Kan	This study
JC351	MG1655 Δ*wzzE* Δ*wzzB* Δ*fepE*	This study
JC53	MG1655 Δ*wzzE* p*wzzE*$_{\mathrm{N172D}}$-*cat*	This study
JC54	MG1655 Δ*wzzE* p*wzzE*$_{\mathrm{N173D}}$-*cat*	This study
JC55	MG1655 Δ*wzzE* p*wzzE*$_{\mathrm{H189F}}$-*cat*	This study
JC56	MG1655 Δ*wzzE* p*wzzE*$_{\mathrm{D192L}}$-*cat*	This study
JC60	MG1655 Δ*wzzE* p*wzzE*$_{\mathrm{R185A}}$-*cat*	This study
JC61	MG1655 Δ*wzzE* p*wzzE*$_{\mathrm{R222A}}$-*cat*	This study
JC62	MG1655 Δ*wzzE* p*wzzE*$_{\mathrm{RR222/3AA}}$-*cat*	This study
JC63	MG1655 Δ*wzzE* p*wzzE*$_{\mathrm{RR222/3KK}}$-*cat*	This study
JC28	MG1655 Δ*wzzE* p*wzzE*$_{\mathrm{L175P}}$-*cat*	This study
JC29	MG1655 Δ*wzzE* p*wzzE*$_{\mathrm{L175R}}$-*cat*	This study
JC30	MG1655 Δ*wzzE* p*wzzE*$_{\mathrm{D92V}}$-*cat*	This study
JC31	MG1655 Δ*wzzE* p*wzzE*$_{\mathrm{K132A}}$-*cat*	This study
JC64	MG1655 Δ*wzzE* p*wzzE*$_{\mathrm{M224A}}$-*cat*	This study
JC65	MG1655 Δ*wzzE* p*wzzE*$_{\mathrm{T167V}}$-*cat*	This study
JC80	MG1655 Δ*wzzE* p*wzzE*$_{\mathrm{E225Q}}$-*cat*	This study
JC81	MG1655 Δ*wzzE* p*wzzE*$_{\mathrm{K232A}}$-*cat*	This study
JC82	MG1655 Δ*wzzE* p*wzzE*$_{\mathrm{ALK230\_2SLD}}$-*cat*	This study

Table S3: CRISPR spacer oligos and repair templates

Oligo/Geneblock	Sequence 5'→3'
**ΔF104 Repair Template Gene Block**	atgACACAACCAATGCCTGGGAAACCGGCCGAAGACGCT<br>GAAAATGAACTGGATATTCGTGGGTTGTTTCGTACCTTGTG<br>GGCTGGGAAGCTATGGATTATTGGCATGGGGCTGGCGTT<br>TGCGTTAATCGCGCTGGCGTATACGTTTTTTGCTCGTCAG<br>GAGTGGAGCTCGACGGCGATTACCGATCGTCCAACGGT<br>GAATATGCTGGGGGGATATTACTCGCAGCAGCAATTTTTG<br>CGTAACCTGGATGTCCGTTCAAACATGGCTTCTGCCGAC<br>CAACCATCGGTCATGGACGAAGCCTACAAAGAGGTTATG<br>CAGCTGGCCAGTTGGGATACCCGCAGAGAGTTCTGGCT<br>GCAAACCGACTATTACAAACAGCGGATGGTGGGCAACA<br>GCAAAGCCGATGCGGCGTTGCTGGATGAAATGATTAACA<br>ACATCCAGTTTATCCCCGGAGACTTTACCCGCGCGGTCA<br>ATGACAGCGTGAAGCTTATTGCCGAAACCGCGCCTGAC<br>GCTAATAACCTGTTACGTCAGTATGTTGCTTTTGCCAGCC<br>AGCGTGCAGCCAGCCATCTGAATGATGAGCTGAAAGGC<br>GCATGGGCGGCGCGTACCATCCAGATGAAAGCTCAGGT<br>GAAGCGTCAGGAAGAGGTGGCGAAAGCCATCTACGACC<br>GCCGGATGAACAGCATTGAGCAGGCGCTGAAAATTGCT<br>GAGCAGCATAATATTTCGCGCAGTGCGACAGATGTACCT<br>GCCGAGGAATTA
**F104H Repair Template Gene Block**	atgACACAACCAATGCCTGGGAAACCGGCCGAAGACGCT<br>GAAAATGAACTGGATATTCGTGGGTTGTTTCGTACCTTGTG<br>GGCTGGGAAGCTATGGATTATTGGCATGGGGCTGGCGTT<br>TGCGTTAATCGCGCTGGCGTATACGTTTTTTGCTCGTCAG<br>GAGTGGAGCTCGACGGCGATTACCGATCGTCCAACGGT<br>GAATATGCTGGGGGGATATTACTCGCAGCAGCAATTTTTG<br>CGTAACCTGGATGTCCGTTCAAACATGGCTTCTGCCGAC<br>CAACCATCGGTCATGGACGAAGCCTACAAAGAGCACGTT<br>ATGCAGCTGGCCAGTTGGGATACCCGCAGAGAGTTCTG<br>GCTGCAAACCGACTATTACAAACAGCGGATGGTGGGCAA<br>CAGCAAAGCCGATGCGGCGTTGCTGGATGAAATGATTAA<br>CAACATCCAGTTTATCCCCGGAGACTTTACCCGCGCGGT<br>CAATGACAGCGTGAAGCTTATTGCCGAAACCGCGCCTGA<br>CGCTAATAACCTGTTACGTCAGTATGTTGCTTTTGCCAGC<br>CAGCGTGCAGCCAGCCATCTGAATGATGAGCTGAAAGG<br>CGCATGGGCGGCGCGTACCATCCAGATGAAAGCTCAGG<br>TGAAGCGTCAGGAAGAGGTGGCGAAAGCCATCTACGAC<br>CGCCGGATGAACAGCATTGAGCAGGCGCTGAAAATTGCT

Oligo/Geneblock	Sequence 5'→3'
	GAGCAGCATAATATTTCGCGCAGTGCGACAGATGTACCT<br>GCCGAGGAA
**F104Y Repair Template Gene Block**	atgACACAACCAATGCCTGGGAAACCGGCCGAAGACGCT<br>GAAAATGAACTGGATATTCGTGGGTTGTTTCGTACCTTGTG<br>GGCTGGGAAGCTATGGATTATTGGCATGGGGCTGGCGTT<br>TGCGTTAATCGCGCTGGCGTATACGTTTTTTGCTCGTCAG<br>GAGTGGAGCTCGACGGCGATTACCGATCGTCCAACGGT<br>GAATATGCTGGGGGGATATTACTCGCAGCAGCAATTTTTG<br>CGTAACCTGGATGTCCGTTCAAACATGGCTTCTGCCGAC<br>CAACCATCGGTCATGGACGAAGCCTACAAAGAGTACGTT<br>ATGCAGCTGGCCAGTTGGGATACCCGCAGAGAGTTCTG<br>GCTGCAAACCGACTATTACAAACAGCGGATGGTGGGCAA<br>CAGCAAAGCCGATGCGGCGTTGCTGGATGAAATGATTAA<br>CAACATCCAGTTTATCCCCGGAGACTTTACCCGCGCGGT<br>CAATGACAGCGTGAAGCTTATTGCCGAAACCGCGCCTGA<br>CGCTAATAACCTGTTACGTCAGTATGTTGCTTTTGCCAGC<br>CAGCGTGCAGCCAGCCATCTGAATGATGAGCTGAAAGG<br>CGCATGGGCGGCGCGTACCATCCAGATGAAAGCTCAGG<br>TGAAGCGTCAGGAAGAGGTGGCGAAAGCCATCTACGAC<br>CGCCGGATGAACAGCATTGAGCAGGCGCTGAAAATTGCT<br>GAGCAGCATAATATTTCGCGCAGTGCGACAGATGTACCT<br>GCCGAGGAA
**GG333/4LL Repair Template Gene Block**	ATGACACAACCAATGCCTGGGAAACCGGCCGAAGACGC<br>TGAAAATGAACTGGATATTCGTGGGTTGTTTCGTACCTTGT<br>GGGCTGGGAAGCTATGGATTATTGGCATGGGGCTGGCGT<br>TTGCGTTAATCGCGCTGGCGTATACGTTTTTTGCTCGTCA<br>GGAGTGGAGCTCGACGGCGATTACCGATCGTCCAACGG<br>TGAATATGCTGGGGGGATATTACTCGCAGCAGCAATTTTT<br>GCGTAACCTGGATGTCCGTTCAAACATGGCTTCTGCCGA<br>CCAACCATCGGTCATGGACGAAGCCTATAAAGAGTTTGTT<br>ATGCAACTGGCCTCGTGGGATACCCGCAGAGAGTTCTG<br>GCTGCAAACCGACTATTACAAACAGCGGATGGTGGGCAA<br>CAGCAAAGCCGATGCGGCGTTGCTGGATGAAATGATTAA<br>CAACATCCAGTTTATCCCCGGAGACTTTACCCGCGCGGT<br>CAATGACAGCGTGAAGCTTATTGCCGAAACCGCGCCTGA<br>CGCTAATAACCTGTTACGTCAGTATGTTGCTTTTGCCAGC<br>CAGCGTGCAGCCAGCCATCTGAATGATGAGCTGAAAGG<br>CGCATGGGCGGCGCGTACCATCCAGATGAAAGCTCAGG<br>TGAAGCGTCAGGAAGAGGTGGCGAAAGCCATCTACGAC

Oligo/Geneblock	Sequence 5'→3'
	CGCCGGATGAACAGCATTGAGCAGGCGCTGAAAATTGCT<br>GAGCAGCATAATATTTCGCGCAGTGCGACAGATGTACCT<br>GCCGAGGAATTACCTGATTCAGAAATGTTCCTGCTTGGG<br>CGTCCAATGCTTCAGGCTCGACTGGAAAATTTACAGGCC<br>GTCGGTCCGGCCTTTGATCTCGACTATGATCAGAATCGG<br>GCCATGTTAAACACCCTGAATGTTGGTCCAACCCTGGAT<br>CCGCGTTTTCAGACCTATCGCTATTTGCGTACGCCGGAA<br>GAACCGGTAAAACGCGATAGCCCACGTCGTGCCTTCCT<br>GATGATTATGTGGGGCATTGTCcttcttCTGATCGGGGCTG<br>GTGTCGCATTAACCCGCCGTTGCTCGAAATAG
**F104 Protospacer Fwd**	AAACTATAAAGAGTTTGTTATGCAACTGGCCTCGG
**F104 Protospacer Rvr**	AAAACCGAGGCCAGTTGCATAACAAACTCTTTATA
**GG333/4LL Protopacer Fwd**	AAACTTCCTGATGATTATGTGGGGCATTGTCGGGG
**GG333/4LL protospacer Rvr**	AAAACCCCGACAATGCCCCACATAATCATCAGGAA
**wzzE_x3F Gene Block**	GGTGTCGCATTCCGCCGTTGCTCGAAAGGAGGCAGCGG<br>TGGGAGTGGCGGAGACTACAAGGACCACGACGGTGACT<br>ACAAGGACCACGACATCGACTACAAGGACGACGACGAC<br>AAGTGRAAAGTGGATCCGTCGACCTGCAGCCA

Table S4: Primers used in this study

Primer	Sequence 5' → 3'
pZS21_fwd	agtggatccgtcgacctg
pZS21_rev	catggtacctttctttaatgaattcgg
pZS21-wzzE_fwd	cattaaagaaaggtaccatggcgtagaaatcgtggtgg
wzzE-pZS21_rev	tgcaggtcgacggatccactttatttcgagcaacggcgg
wzzE-cat_rev	ttttctccattttcgagcaacggcgggt
wzzE-cat_fwd	ttgctcgaaaatggagaaaaaaatcactggatataccaccgttgatatatccc
cat-pZS21_rev	tgcaggtcgacggatccactttacgccccgccctgcca
pZS21-wzzE_fwd ΔF104	GTTATGCAACTGGCCTCG
pZS21-wzzE_Rvr ΔF104	CTCTTTATAGGCTTCGTCC
pZS21-wzzE_fwd F104H	CTATAAAGAGcacGTTATGCAACTGGCCTC
pZS21-wzzE_Rvr F104H	GCTTCGTCCATGACCGAT
pZS21-wzzE_fwd F104Y	CTATAAAGAGtatGTTATGCAACTGG
pZS21-wzzE_Rvr F104Y	GCTTCGTCCATGACCGAT
pZS21-wzzE_fwd K162A	TGACAGCGTGgctCTTATTGCCGAAACCG
pZS21-wzzE_Rvr K162A	TTGACCGCGCGGGTAAAG
pZS21-wzzE_fwd K162S	TGACAGCGTGtccCTTATTGCCGAAACCG
pZS21-wzzE_Rvr K162S	TTGACCGCGCGGGTAAAG
pZS21-wzzE_fwd K162T	TGACAGCGTGactCTTATTGCCGAAACCGC
pZS21-wzzE_Rvr K162T	TTGACCGCGCGGGTAAAG
pZS21-wzzE_fwd L259A	AATGTTCCTGgctGGGCGTCCAATG
pZS21-wzzE_Rvr L259A	TCTGAATCAGGTAATTCCTC
pZS21-wzzE_Rvr L259I	TCTGAATCAGGTAATTCCTC
pZS21-wzzE_Fwd L259I	AATGTTCCTGattGGGCGTCCAA
pZS21-wzzE_fwd N172D	GCCTGACGCTgacAACCTGTTAC
pZS21-wzzE_Rvr N172D	GCGGTTTCGGCAATAAGC
pZS21-wzzE_fwd N173D	TGACGCTAATgatCTGTTACGTCAGTATGTTG
pZS21-wzzE_Rvr N173D	GCGGTTTCGGCAATAAGC
pZS21-wzzE_fwd H189F	TGCAGCCAGCttcCTGAATGATGAGCTGAAAG
pZS21-wzzE_Rvr H189F	CGCTGGCTGGCAAAAGCA
pZS21-wzzE_fwd D192L	CCATCTGAATcttGAGCTGAAAGGCGCATGG
pZS21-wzzE_Rvr D192L	CTGGCTGCACGCTGGCTG
pZS21-wzzE_fwd M224A	CGACCGCCGGgccAACAGCATTG
pZS21-wzzE_Rvr M224A	TAGATGGCTTTCGCCACC
pZS21-wzzE_fwd R185A	TGCCAGCCAGgccGCAGCCAGCC
pZS21-wzzE_Rvr R185A	AAAGCAACATACTGACGTAACAG
pZS21-wzzE_fwd RR222/3AA	CATCTACGACgccgccATGAACAGCATTGAG
pZS21-wzzE_Rvr RR222/3AA	GCTTTCGCCACCTCTTCC
pZS21-wzzE_fwd RR222/3KK	CATCTACGACaaaaaaATGAACAGCATTGAGCAG

**Primer**	**Sequence 5' → 3'**
pZS21-wzzE_Rvr RR222/3KK	GCTTTCGCCACCTCTTCC
pZS21-wzzE_fwd L175P	TAATAACCTGcctCGTCAGTATGTTGCTTTTGCCAGCC
pZS21-wzzE_Rvr L175P	GCGTCAGGCGCGGTTTCG
pZS21-wzzE_fwd L175R	TAATAACCTGcgtCGTCAGTATGTTGCTTTTGCCAGCC
pZS21-wzzE_Rvr L175R	GCGTCAGGCGCGGTTTCG
pZS21-wzzE_fwd R241A	TAATATTTCGgccAGTGCGACAGATG
pZS21-wzzE_Rvr R241A	TGCTGCTCAGCAATTTTC
pZS21-wzzE_fwd R222A	CATCTACGACgccCGGATGAACAGCATTG
pZS21-wzzE_Rvr R222A	GCTTTCGCCACCTCTTCC
pZS21-wzzE_fwd T167V	TATTGCCGAAgttGCGCCTGACG
pZS21-wzzE_Rvr T167V	AGCTTCACGCTGTCATTG
pZS21-wzzE_fwd D92V	GGCTTCTGCCgttCAACCATCGG
pZS21-wzzE_Rvr D92V	ATGTTTGAACGGACATCC
pZS21-wzzE_fwd E255Q	ACCTGATTCAcaaATGTTCCTGCTTG
pZS21-wzzE_Rvr E255Q	AATTCCTCGGCAGGTACA
pZS21-wzzE_fwd K232A	GCAGGCGCTGgccATTGCTGAGC
pZS21-wzzE_Rvr K232A	TCAATGCTGTTCATCCG
pZS21-wzzE_fwd ALK230_2SLD	agacATTGCTGAGCAGCATAATATTTC
pZS21-wzzE_Rvr ALK230_2SLD	aaggaCTGCTCAATGCTGTTCATC
pZS21-wzzE_fwd A323G	CCCACGTCGTggtTTCCTGATGATTATG
pZS21-wzzE_Rvr A323G	CTATCGCGTTTTACCGGT
pZS21-wzzE_fwd GG333/4AA	GGGCATTGTCgctgctCTGATCGGGG
pZS21-wzzE_Rvr GG333/4AA	CACATAATCATCAGGAAGG
pZS21-wzzE_fwd GG333/4LL	GGGCATTGTCcttcttCTGATCGGGG
pZS21-wzzE_Rvr GG333/4LL	CACATAATCATCAGGAAGG
pZS21-wzzE_fwd G339A	GATCGGGGCTgctGTCGCATTAACCC
pZS21-wzzE_Rvr G339A	AGCCCCCCGACAATGCCC
pwzzE_x3F Fwd	TGATAAGTGGATCCGTCGA
pwzzE_x3F Rvr	ATTGGGCGGCAACGAGCTTT
WzzE: Cmr Fp	TAACCCGCCGTTGCTCGAAAATGGAGAAAAAAATCACT GG
WzzE: Cmr Rp	CCACTCCCACCGCTGCCTCCCGCCCCGCCCTGCCA CTCAT
Cmr: GS-x3F FP	GGAGGCAGCGGTGGGAGTGGCGGAGACTACAAGGA CCACGA

SUPPLEMENTAL DATASETS

Data Set S1: Ion fragmentation data for LC-MS/MS of ECA_{CYC}

SUPPLEMENTAL REFERENCES

1. Wiseman B, Widmalm G, Högbom M. 2023. Alternating L4 loop architecture of the bacterial polysaccharide co-polymerase WzzE. Commun Biol 6:802.
2. Färnbäck M, Eriksson L, Senchenkova Sy, Zych K, Knirel YA, Sidorczyk Z, Widmalm G. 2003. Crystal structure of a cyclic enterobacterial common antigen. Angewandte Chemie 42:2543-2546.
3. Abramson J, Adler J, Dunger J, Evans R, Green T, Pritzel A, Ronneberger O, Willmore L, Ballard AJ, Bambrick J, Bodenstein SW, Evans DA, Hung C-C, O'Neill M, Reiman D, Tunyasuvunakool K, Wu Z, Žemgulytė A, Arvaniti E, Beattie C, Bertolli O, Bridgland A, Cherepanov A, Congreve M, Cowen-Rivers AI, Cowie A, Figurnov M, Fuchs FB, Gladman H, Jain R, Khan YA, Low CMR, Perlin K, Potapenko A, Savy P, Singh S, Stecula A, Thillaisundaram A, Tong C, Yakneen S, Zhong ED, Zielinski M, Žídek A, Bapst V, Kohli P, Jaderberg M, Hassabis D, Jumper JM. 2024. Accurate structure prediction of biomolecular interactions with AlphaFold 3. Nature 630:493-500.
4. Berlyn MKB. 1999. CGSC: The E.coli Genetic Stock Center Database, p 175-183. *In* Letovsky S (ed), Bioinformatics: Databases and Systems doi:10.1007/0-306-469030_16. Springer US, Boston, MA.
5. Mitchell AM, Srikumar T, Silhavy TJ. 2018. Cyclic Enterobacterial Common Antigen Maintains the Outer Membrane Permeability Barrier of Escherichia coli in a Manner Controlled by YhdP. mBio 9.
